# Establishment of a human induced pluripotent stem cell neuronal model for identification of modulators of A53T α-synuclein levels and aggregation

**DOI:** 10.1371/journal.pone.0261536

**Published:** 2021-12-21

**Authors:** Charlotte Vajhøj, Benjamin Schmid, Ania Alik, Ronald Melki, Karina Fog, Bjørn Holst, Tina Charlotte Stummann

**Affiliations:** 1 H. Lundbeck A/S, Valby, Denmark; 2 Bioneer A/S, Hørsholm, Denmark; 3 Institute Francois Jacob (MIRCen), CEA and Laboratory of Neurodegenerative Diseases, CNRS, Fontenay-Aux-Roses cedex, France; Louisiana State University Health Sciences Center, UNITED STATES

## Abstract

Inhibiting formation or promoting degradation of α-synuclein aggregates are among the therapeutical approaches under investigation as disease-modifying treatment strategies for Parkinson’s disease. To support these developments, several *in vitro* models based on seeded α-synuclein aggregation have been established in immortalized cell lines and murine primary neurons. Here, we report on a humanized model with a reproducibility and throughput that enables its use in supporting target identification and validation in pharmacological research. A human induced pluripotent stem cell (iPSC) line was genetically modified to express HA-tagged α-synuclein with the point mutation in position 53 from Alanine to Threonine (A53T) under an inducible system and differentiated into cortical neurons expressing neuronal markers and exhibiting spontaneous activity. Intracellular α-synuclein aggregation was triggered by exposure to exogenous added fibrillated recombinant wild-type human α-synuclein fibrils91 and demonstrated by several endpoints; the formation of Triton-insoluble SDS-soluble α-synuclein, biochemically in a fluorescence resonance energy transfer based aggregation assay and by immunocytochemistry of phosphorylated α-synuclein positive puncta. We demonstrate the feasibility of upscaling the iPSC neuron production for drug discovery and that the model has a suitable dynamic range allowing for both detection of increased and decreased α-synuclein aggregation. Moreover, gene modulation is feasible using siRNAs, making the model suitable for genetic screening for modulators of α-synuclein aggregation. Data on effects of USP8, USP13 and USP9X knockdown on α-synuclein expression and aggregation contradicts published data from immortalized cell lines and murine systems. This highlight the importance of including humanized neuronal models in the confirmation of biological mechanisms in specific variations of Parkinson’s disease.

## Introduction

Targeting α-synuclein is currently one of the most pursued disease-modifying treatment strategies for Parkinson’s disease. Toxic α-synuclein species have been shown to disrupt important cellular functions, and inhibiting formation or promoting degradation of α-synuclein aggregates are among the therapeutical approaches under investigation [1]. The development of cellular models, where addition of exogenous pathological α-synuclein fibrils to immortalized cell lines or murine primary neurons leads to misfolding, aggregation and phosphorylation of endogenous α-synuclein, has provided valuable model systems capturing key pathological phenotypes of Parkinson’s disease [2–4].

Models based on dopaminergic neurons derived from Parkinson’s patient iPSCs have demonstrated higher α-synuclein protein levels and increased phosphorylation of α-synuclein at serine residue 129 (pS129) compared to controls [5, 6]. The limited reports on spontaneous aggregation in these models show data on colocalization of Thioflavin T with α-synuclein and the presence of Triton insoluble α-synuclein [6–9]. The robustness of the findings remains to be confirmed.

Recently, recombinant α-synuclein fibrils have been demonstrated to increase endogenous pS129 α-synuclein and aggregation in human iPSC cortical neurons from healthy individuals [10, 11]. Aggregation of α-synuclein in the iPSC models was shown by assessment of Triton insoluble α-synuclein by fractionation, an assay with a very low throughput, by the use of Thioflavin T, exhibiting enhanced fluorescence upon binding to amyloid fibrils and hence is not specific for α-synuclein, and by immunostaining for pS129 α-synuclein as proxy maker for aggregation. Assays quantifying the α-synuclein aggregation directly would be preferable in the search for therapeutical approaches modulating this feature of Parkinson’s disease. In this paper, we take advantage of gene-editing to establish an iPSC lines with doxycycline inducible HA-tagged α-synuclein A53T expression, exogenous fibrils to seed intracellular α-synuclein aggregation and a fluorescence resonance energy transfer (FRET)-based α-synuclein aggregation assay. Together, this enabled us to develop a robust and reliable human neuronal model suitable for drug discovery genetic screens for modulators of α-synuclein aggregation.

## Materials and methods

### iPSC generation and culturing

The derivation of the iPSC line BIONi010-C from an apparently healthy 18 years old male has previously been described [12]. Gene-editing was carried out using TALEN technology to generate the BIONi010-C-24 iPSC line used in the present study. The cDNA sequence of the SNCA construct with A53T mutation and a single HA-tag (ATGGATGTATTCATGAAAGGACTTTCAAAGGCCAAGGAGGGAGTTGTGGCTGCTGCTGAGAAAACCAAACAGGGTGTGGCAGAAGCAGCAGGAAAGACAAAAGAGGGTGTTCTCTATGTAGGCTCCAAAACCAAGGAGGGAGTGGTGCATGGTGTG**A**CAACAGTGGCTGAGAAGACCAAAGAGCAAGTGACAAATGTTGGAGGAGCAGTGGTGACGGGTGTGACAGCAGTAGCCCAGAAGACAGTGGAGGGAGCAGGGAGCATTGCAGCAGCCACTGGCTTTGTCAAAAAGGACCAGTTGGGCAAGAATGAAGAAGGAGCCCCACAGGAAGGAATTCTGGAAGATATGCCTGTGGATCCTGACAATGAGGCTTATGAAATGCCTTCTGAGGAAGGGTATCAAGACTACGAACCTGAAGCC*TACCCATACGATGTTCCAGATTACGCT*TAA, A53T mutation in bold and underlined, HA-tag in italic and underlined) was designed with an N-terminal NcoI site and a C-terminal KpnI site. A plasmid, containing the two homology arms, a neomycin (Neo) resistance gene and a tetracycline/doxycycline responsive element, was used as backbone vector for the SNCA-HA construct (TRE-TIGHT-EGFP-backward donor, a gift from Rudolf Jaenisch corresponding to Addgene plasmid # 22077; http://n2t.net/addgene:22077; RRID:Addgene_22077). The SNCA-HA cDNA was cloned into this vector using the two mentioned restriction enzymes. To make the line inducible for doxycycline, a plasmid with the reverse TET transactivator (m2rtTA) was used (AAVS1-Neo-M2rtTA, a gift from Rudolf Jaenisch corresponding to Addgene plasmid # 60843; http://n2t.net/addgene:60843; RRID:Addgene_60843). The m2rtTA plasmid contained a puromycine (Puro) resistance gene enabling double selection for Neo and Puro. To target the AAVS1 locus, two TALENs were used (AAVS1-TALEN-L, a gift from Danwei Huangfu corresponding to Addgene plasmid # 59025; http://n2t.net/addgene:59025; RRID:Addgene_59025 and AAVS1-TALEN-R, a gift from Danwei Huangfu corresponding to Addgene plasmid # 59026; http://n2t.net/addgene:59026; RRID:Addgene_59026).

The iPSCs were cultured in E8 medium on Matrigel (Corning Bioscience) coated 6w-dishes and detached using Accutase. When they reached a density of 70–90%, a total of 1.5 million cells were co-nucleofected with 2 μg of both TALEN plasmids, 2 μg of the plasmid containing the m2rtTA and 2 μg of the SNCA-HA plasmid. For the nucleofection, the P3 Primary Cell Kit (Lonza) was used and run with the program CA167 following to the manufacturer’s instructions. The nuclofected iPSCs were transferred back to a Matrigel-coated 100 mm dish in E8 medium supplemented with 1:200 diluted Revita cell supplement (Gibco). 24 h post-nucleofection, cells were subjected to double selection with 1 μg/mL puromycin (Invitrogen) for 2 days and 250 μg/mL neomycin (Gibco) for 4 days. After selection, cells were allowed to recover for one week. Resistant colonies were then picked and expanded. DNA from the clones was extracted and a PCR was run to check for correct integration of the plasmids into the AAVS1 locus using primer pairs specific for the M2rtTA-plasmid (ccagaggcggcgcagaagccag and atcttgttcaatggccgatcccat) and the SNCA-HA-plasmid (ccagaggcggcgcagaagccag and ccgtgggcttgtactcggtcat). The BIONi010-C-24 lines is commercially available from the EBiSC collection (European Collection of Authenticated Cell Cultures).

The BIONi010-C-24 iPSCs were cultured in Essential 8 (Gibco A15169-01) media on Matrigel (BD 354277) coat 6w-dishes and split with EDTA (Ambion AM9206G). We generated mother stock vials frozen in nitrogen tanks. From each of these, we generate a large bank of iPSC vials frozen at 140°C to be used for starting new differentiation. In practice, this enables differentiating to always start from the same passage number.

### α-synuclein fibrillar assemblies

Recombinant wild-type human α-synuclein was purified and assembled into the fibrillar polymorphs fibrils91 as previously described [13–15]. Assembly was monitored using Thioflavin T as previously described [16]. Monomeric alpha-synuclein concentration was determined spectrophotometrically using the extinction coefficients 5960 M^-1^∙cm^-1^ at 280 nm. Fibrillar assembly concentration was determined by subtracting the concentration of α-synuclein in the supernatant fraction from the initial protein concentration after centrifugation at 100,000 g for 30 min. The resuspended fibrils91 (in PBS) were fragmented prior to addition to cell cultures by sonication for 20 min in 2 mL Eppendorf tubes using a Vial Tweeter powered by ultrasonic processor UIS250v (250W, 2.4kHz; Hielscher Ultrasonic, Teltow, Germany), aliquoted, flash frozen in liquid nitrogen and stored until use at -80°C. Quality control was performed before and after: Fibrils91 were imaged by TEM after negative staining with Uranyl acetate using a Jeol 1400 transmission electron microscope after adsorption on 200 mesh, carbon coated, electron micrograph grids. The images were recorded using a Gatan Orius CCD camera (Gatan Inc. Pleasanton, CA, USA). Fibrils91 (1.4 mg/ml equivalent monomer concentration) were also digested at 37°C in PBS with Proteinase K (3.8 ug/ml) (Roche). Aliquots were removed at different time intervals following addition of the protease (0, 1, 5, 15, 30 and 60 minutes) and transferred into Eppendorf tubes with proteinase K inhibitors (phenylmethylsulfonyl fluoride). The samples where dried using speed vacuum and further solubilized by addition of pure Hexafluoroisopropanol (HFIP). After evaporation of HFIP, the samples were resuspended in laemmli buffer, heated 10 minutes at 70°C and analyzed by Tris-Glycine SDS-PAGE (15%) after Coomassie blue staining.

### iPSC neuronal differentiation, α-synuclein seeding and siRNA exposure

A modified version of a previously published neuronal differentiation protocol was used [17]. Differentiation was initiated day 0 by dual SMAD inhibition (100 nM LDN193189 (Sigma-Aldrich SML0559) and 10 μM SB431542 (Sigma-Aldrich S4317)) in N3 media (50% DMEM-F12 (Thermo Fisher Scientific 31331–028), 50% Neurobasal medium (Thermo Fisher Scientific 21103–049), 0.5% N2 (Thermo Fisher Scientific, 17502–048), 0.5% B27 (Thermo Fisher Scientific 17504–044), 0.5 mM GlutaMAX supplement (Thermo Fisher Scientific 35050–061), 0.5% NEAA supplement (Thermo Fisher Scientific 11140–050), 50 μM 2-mercaptoethanol (Thermo Fisher Scientific 21985023), 2.5 μg/mL insulin (Sigma-Aldrich 19278)) of 500,000 iPSCs per cm^2^ plated on Matrigel coated dishes. The cells were split at day 7, 12 and 17 and re-plated as on day 0, except that from day 12 and onwards poly-L-ornithine/laminin coat was used and that LDN193189 and SB431542 were withdrawn from day 13. The neuronal progenitor cells were frozen day 21 to generate a neuronal progenitor bank. Upon thawing the neuronal progenitors were allowed to proliferate for 4 days, plated day 25 and subjecting to neuronal maturation medium (N3 with 20 ng/mL BDNF (R&D Systems 248-BD), 10 ng/mL GDNF (R&D Systems 212-GD), 200 μM L-Ascorbic Acid (Sigma-Aldrich A5960), and 500 μM db-cAMP (Sigma-Aldrich D0627) from day 26 and onwards. Medium containing maturation factors was used within a week and changed every 2–3 days. Day 32, the cells were re-plated into assay plates at a density of 167,000 cells/cm^2^, except that 333,000 cells/cm^2^ were used for measurement of spontaneous calcium oscillation. 96 well formats were used for all assays except that 6 wells were used for fractionation and western blot samples. At all timepoints of differentiation, cells were split using accutase (Thermo Fisher Scientific A11105) and 10 μM ROCK inhibitor (Sigma-Aldrich Y0503) was added overnight upon thawing and splitting. On day 33, doxycycline (1 μg/mL unless otherwise indicated, Stemgent #04–0016) was added to the cultures to induce expression of HA-tagged A53T α-synuclein. Horizon Discovery Accell siRNAs (1 μM) were added day 36 and 43 and left on for 72h before the media was refreshed. Pools of four different siRNAs were used as non-targeting control (D-001910-10) and to target USP8 (E-005203-00), USP13 (E-006064-00) and USP9X (E-006099-00). Custom made siRNAs were used for SCNA (sense sequences for SCNA siRNA_1, 2 and 3 were GAUGUAUUCAUGAAAGGACUU, GGGUGUUCUCUAUGUAGGCUU and GAGCAAGUGACAAAUGUUGUU). On day 46, fragmented recombinant full-length wild-type human α-synuclein fibrils91 were thawed and immediately added to the iPSC neurons. The fibrils were left on for 96h before changing the media. Cells were harvest for analysis on day 60 unless indicated otherwise.

### Real-time quantitative polymerase chain reaction (RT-qPCR)

SYBR Green Cell-to-Ct kit (Ambion 4402957) was used for RNA isolation and reverse transcription to cDNA. RT-qPCR was performed with TaqMan^®^ Fast Advanced Master Mix and TaqMan primers purchased from Thermo Scientific. The 2^-ΔΔCt^ method was applied to expression data and genes of interests (POU5F1 Hs04260367_gH, TUBB3 Hs00801390_s1, MAPT Hs00902190_m1, MAP2 Hs00258900_m1, RBFOX3 Hs01370653_m1, CAMK2A Hs00947041_m1, SLC17A7 Hs00220404_m1, SLC17A6 Hs00220439_m1, GAD1 Hs01065892_m1, SLC6A1 Hs01104475_m1, SLC6A11 Hs01117194_m1, SLC32A1 Hs00369773_m1, SNCA Hs01103383_m1) were normalized to geometric mean of housekeeping genes (GUSB Hs00939627_m1, RPL13A Hs04194366_g1, HSP90AB1 Hs04194340_g1) and mean of undifferentiated iPSC cDNA samples.

### Immunocytochemistry

Cells were fixed in 4% paraformaldehyde for 15 min at room temperature, blocked 20 min in KPBS (10 mM phosphate buffer, 150 mM NaCl, and 3.4 mM KCl) supplemented with 0.5% BSA, 0.1% Triton X-100, and 5% normal swine serum (Jackson ImmunoResearch, 014-000-121) and incubated overnight at 4°C in blocking buffer containing primary antibody (Oct-4 1:500 Stemgent 09–0023, Nestin 1:5000 Millipore MAB5326, Pax6 1:1000 BioLegend 901301, Ki67 1:200 Millipore MAB4190, βIII-Tubulin 1:200 GeneTex GTX631836, total tau 1:1000 Dako A0024, synapsin-I 1:200 Abcam ab64581, GFAP 1:2000 Dako z0334, GABA 1:1000 Sigma A2052, Doublecortin 1:200 Abcam ab18723, pS129 α-synuclein 1:500 Abcam ab51253, HA-tag 1:1000 Santa Cruz sc-7392, MAP2 1:5000 Abcam ab5392). Subsequently, they were incubated with secondary antibodies (Alexa Fluor Secondary Antibodies from Invitrogen) and Hoechst 34580 (BDbiosciences 565877) for 1 hour at room temperature, mounted in Prolong Gold Antifade Mountant (Thermo Scientific, P36934), and visualized on Leica DM5500 B fluorescent microscope (Leica, DE) or using a Nikon Eclipse Ti 4.10 microscope combined with a Yokogawa CSU-X1 spinning disk confocal scanner and a Hamamatsu UltraVIEW VoX C9100-50 EMCCD camera.

### Intracellular calcium measurements

Neurons were incubated for 1h with the fluorescent calcium indicator FLIPR Calcium 5 (Molecular Devices R8186) diluted 3x more than providers recommendation in Hanks balanced salt solution (ThermoFisher Scientific, 14175) with 20 mM Hepes and 1.26 mM CaCl2. Image based measurements of cytosolic calcium kinetics were conducted at 37°C and with a 1 Hz sampling frequency (excitation and emission wavelengths of 480, and 540 nm, respectively) using the FDSS7000 system (Hamamatsu, Japan). The instrument readout is the total fluorescent intensity per well and data are reported as ratio to the first sampled data point.

### Fractionation and western blotting

Whole cell lysates were generated by lysis for 30 min in FNN0011 buffer (ThermoFisher Scientific) supplemented with protease and phosphatase inhibitor (Roche, 11697498001, 04906837001). Lysate was centrifuged (18.000xg, 20 min, 4°C), and the supernatant was collected and stored at -80°C until western blotting. Fractionation samples were generated by lysing the cells from a 6-well in 100μl 1% Triton X-100 in 50 mM Tris, 150 mM NaCl (pH 7.6) with protease and phosphatase inhibitor (Roche, 11697498001, 04906837001) for 15 min on ice followed by sonication using a Branson Sonifier 250. Ultracentrifuge at 100.000g for 30 min at 4 °C resulted in the “Triton fraction” supernatant. The pellet was washed once by adding and removing buffer again without dissolving pellet, resuspend in 100μl SDS buffer (1% SDS in 50 mM Tris, 150 mM NaCl (pH 7.6) with protease and phosphatase inhibitor), sonicated and ultra-centrifuged at 100.000g for 30 min at RT. This supernatant was the “SDS fraction”. Both supernatants were frozen at -80°C until further processing. 15 μl of the fractions were loaded per well of the polyacrylamide gels. All western blots were performed with standard methods. Recombinant tau protein ladder was included in some blots (Sigma T7951). Nitrocellulose membranes were placed in 98°C PBS for 5 min before blotting with α-synuclein antibodies for full epitope retrieval. Primary antibodies were βIII-Tubulin 1:400 GeneTex GTX631836, total tau 1:1000 Dako A0024, synapsin-I 1:500 Abcam ab64581, β-actin 1:50.000 Sigma A5441, GAPDH 1:1000 Abcam ab9485, pS129 α-synuclein 1:2000 Abcam ab51253, HA-tag Abcam ab9110, total α-synuclein 1:5000 Thermo Fisher MA1-90346-4B12, USP13 1:500 Proteintech 16840-1-AP, USP8 1:250 Cell Signaling 11832, USP9X 1:500 Cell Signaling 365353. Secondary antibodies were Alexa Flour 680 from Invitrogen or 800CW IRDye from LI-COR. Blots were scanned on a LI-COR Odyssey CLx Instrument and data extracted using LI-COR Imaging Studio software.

### pS129-synuclein and pS129-pS129 aggregation α-synuclein FRET assays

Neurons were washed twice in PBS, before sucking dry and freezing at -80°C until further analysis of the samples. Analysis was performed with commercially available time-resolved fluorescence resonance energy transfer (FRET) kits from Cisbio and a PHERAstar Microplate Reader (BMG LABTECH). One kit was the pS129 α-synuclein assay that is based on a total α-synuclein antibody and a pS129 α-synuclein antibody (Cisbio #6FSYNPEG), detecting only phosphorylated α-synuclein. The other was the pS129-pS129 aggregation α-synuclein assay that is based on one pS129-synuclein antibody labelled either with FRET donor or with acceptor (Cisbio #6FCUS000, please contact Delphine.Jaga@PERKINELMER.COM to get access to this kit), detecting only phosphorylated oligomers or larger α-synuclein assemblies.

### Cell viability assays and total α-synuclein quantification

Cell viability was determined according to the manufacture’s instruction using CellTiter-Glo Luminescent Cell viability assay quantifying the cellular ATP content (Promega G7570). Alternative, resazurin metabolism was used to determine viability by incubating the neurons for 1h at 37°C in neuronal maturation medium containing 10μg/ml resazurin sodium salt (Sigma R7017) and subsequently measuring the fluorescence with excitation wavelength at 530-560nm and emission wavelength at 590nm using an Envision 2103 Multilabel Reader. Total α-synuclein protein quantification was determined according to the manufacture’s instruction using human α-synuclein electrochemiluminescence immunoassay kit (Meso Scale Diagnostics K151TGD).

### Curve fitting and statistical analysis

Arithmetic was conducted in Excel (Microsoft) and curve fitting and statistical analysis in Prism GraphPad. Graphs are shown as mean±SEM (“n” = independent neuronal differentiations, “m” = technical replicates).

## Results

### Establishment of a doxycycline inducible A53T α-synuclein expressing iPSC line and its differentiation into spontaneously active neurons

**Fig 1:**
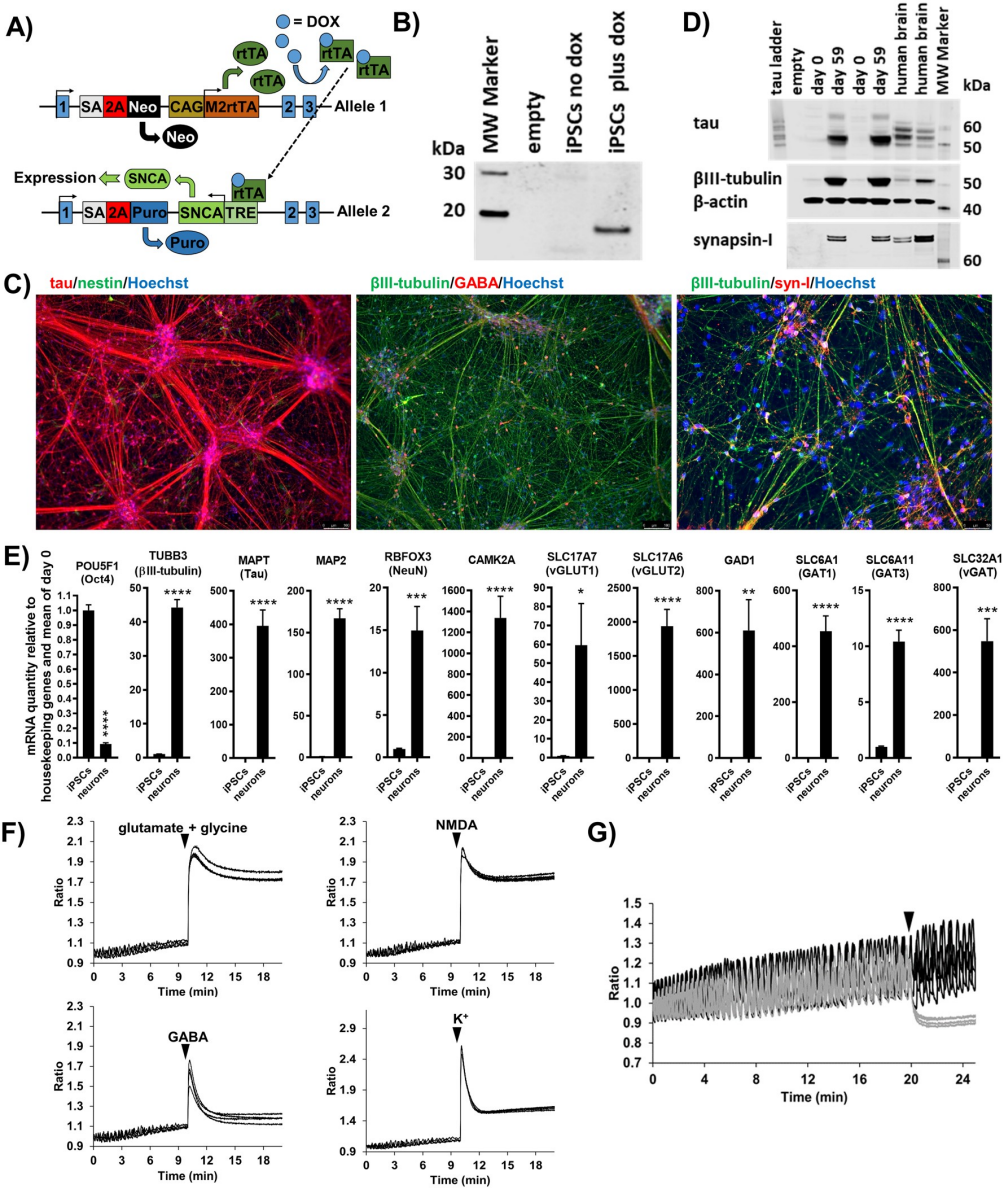
Establishment and differentiation into neurons of iPSC line BIONi010-C-24 with doxycycline inducible A53T α-synuclein. (A) Illustration of the doxycycline (DOX) inducible SNCA expression system: One allele contained the m2 version of the reverse TET transactivator (M2rtTA) under regulation of the CAG promoter. When doxycycline binds the tetracycline reverse transactivator (rtTA), its 3D structure changes facilitating binding to the tetracycline responsive element (TRE) leading to the expression of SNCA-A53T-HA. Neomycin (Neo) and puromycine (Puro) resistance genes enable double selection. SA = splice acceptor, 2A = self-splicing protein. (B) Western Blot analysis for total α-synuclein in pluripotent iPSCs exposed to doxycycline for 2 days showed that the protein was expressed in an inducible manner. (C) Immunocytochemistry of iPSC neurons (day 60 of differentiation) showing expression of the pan-neuronal filament markers tau and βIII-tubulin and the synaptic protein synapsin-I in most cells. A subpopulation of GABA positive cells was present as well as a few cells positive for the neuroprogenitor markers nestin. Scale bars. 200 μm. Representative images from three independent neuronal differentiations. (D) Western Blot analysis for tau, βIII-tubulin and synapsin-I expression in undifferentiated iPSCs (day 0) and iPSC neurons (day 59 of differentiation). Representative blots of three independent neuronal differentiations. (E) RT-qPCR for gene expression of pluripotency marker POU5F1 (Oct-4), pan-neuronal markers TUBB3 (βIII-tubulin), MAPT (tau), MAP2 (microtubule associated protein-2) and RBFOX3 (NeuN), glutamatergic markers CAMK2A, SLC17A7, SLC17A6 and GABAergic markers GAD1, SLC6A1, SLC6A11 and SLC32A1 in iPSC neurons (day 60 of differentiation) versus undifferentiated iPSCs (day 0). Data are mean±SEM (n = 3, m = 4) and analyzed by Students unpaired t-test, ns. non-significant, *p<0.05, **p<0.01, ***p<0.001 and ****p<0.0001. (F) Changes in intracellular calcium in iPSC neurons (day 52/53 of differentiation) following application of the neurotransmitters, glutamate (300 μM glutamate +10 μM glycine), NMDA (40 μM) and GABA (100 μM), or depolarization of the membrane potential by addition of extracellular potassium chloride (25 mM). Representative traces are shown. (G) Spontaneous calcium oscillations in iPSC neurons (day 59/60 of differentiation) were eliminated by addition (timepoint marked with triangle) of 1 μM tetrodotoxin (grey traces) but not buffer (black traces). Representative traces are shown.

We generated the iPSC line BIONi010-C-24 with doxycycline inducible expression of A53T α-synuclein carrying the disease-causing mutations A53T and a HA-tag. We decided for A53T α-synuclein, which is more aggregation prone than wildtype α-synuclein [18], to support the establishment of an α-synuclein seeding with a robust assay window. The HA-tag was added to be able to differentiate endogenously formed fibrils from the seed material. The line was made by inserting two constructs into the AAVS1 safe locus site: One allele contained the SNCA-A53T-HA with a tetracycline responsive element and the other allele contained the reverse TET transactivator (Fig 1A). Addition of doxycycline to the pluripotent iPSCs confirmed inducible expression of HA-tagged A53T α-synuclein (Fig 1B and S1 Fig). Quality control experiments demonstrated purity, normal karyotype and pluripotency of the line (S1 Fig).

Neuronal differentiation of the BIONi010-C-24 line was induced following a modified version of a protocol generating dorsal forebrain cortical neurons [15]. Immunocytochemistry staining of cells on day 0, 12 and 25 of neuronal induction showed Oct4 protein expression in undifferentiated cells that was downregulated upon neuronal induction (S2 Fig). Most cells were positive for the neuronal progenitor markers nestin and the proliferation marker Ki67 on day 25 (S2 Fig), while only a very small cell population was positive on day 60 (Fig 1C and S3 Fig). Pax6, playing a role in dorsal forebrain regionalization, showed a transient protein expression (S2 Fig) reflecting its *in vivo* expression pattern. The neuronal differentiation resulted in cultures that exhibited clear neuronal morphology with extensive outgrowth of neurites (Fig 1C and S3 Fig). Immunocytochemistry of the neuronal cultures at day 45 and 60 confirmed the presence of mature pan-neuronal markers βIII-tubulin, tau, doublecortin, synapsin-I and GABA (Fig 1C and S3 Fig). The majority of the day 45 and 60 cells were positive for the mature pan-neuronal markers, while only few nestin positive progenitors could be identified (Fig 1C and S3 Fig). In addition, sporadic cells, positive for the astrocyte marker GFAP could be identified on day 60 (S4 Fig) but never on day 45. Western Blot analysis further confirmed protein expression of βIII-tubulin, tau and synapsin-I and revealed that the expressed tau was the embryonic 0N3R tau isoform (Fig 1D and S1 Raw images).

RT-qPCR gene expression analysis of neurons (day 60 of differentiation) relative to undifferentiated iPSCs (day 0) confirmed the down-regulation of the pluripotency marker Oct4 and showed upregulation of the pan-neuronal markers TUBB3 (βIII-tubulin), MAPT (tau), microtubule associated protein-2 MAP2 and RBFOX3 (NeuN) (Fig 1E). In line with the cortical differentiation protocol applied, the neurons also had up-regulation of glutamatergic markers CAMK2A, SLC17A7, SLC17A6 and GABAergic markers GAD1, SLC6A1, SLC6A11 and SLC32A1 (Fig 1E).

Image based measurement of cytosolic calcium kinetics showed that neurons responded to extracellular potassium chloride induced depolarization of the membrane potential (Fig 1F and S5 Fig). Furthermore, stimulation with the neurotransmitters glutamate (+ glycine), NMDA and GABA resulted in a considerable increase in cytosolic calcium indicating the presence of functional GABAergic and glutamatergic receptors (Fig 1F and S5 Fig). Plating the neurons at high density resulted in robust spontaneous calcium oscillations, which could be eliminated by addition of the voltage-gated sodium channel blocker tetrodotoxin (1 μM) verifying a role for action potentials in facilitating the coordinated calcium oscillations (Fig 1G and S6 Fig).

### De novo generated fibrils91 seed α-synuclein aggregation in iPSC neurons expressing A53T α-synuclein

**Fig 2:**
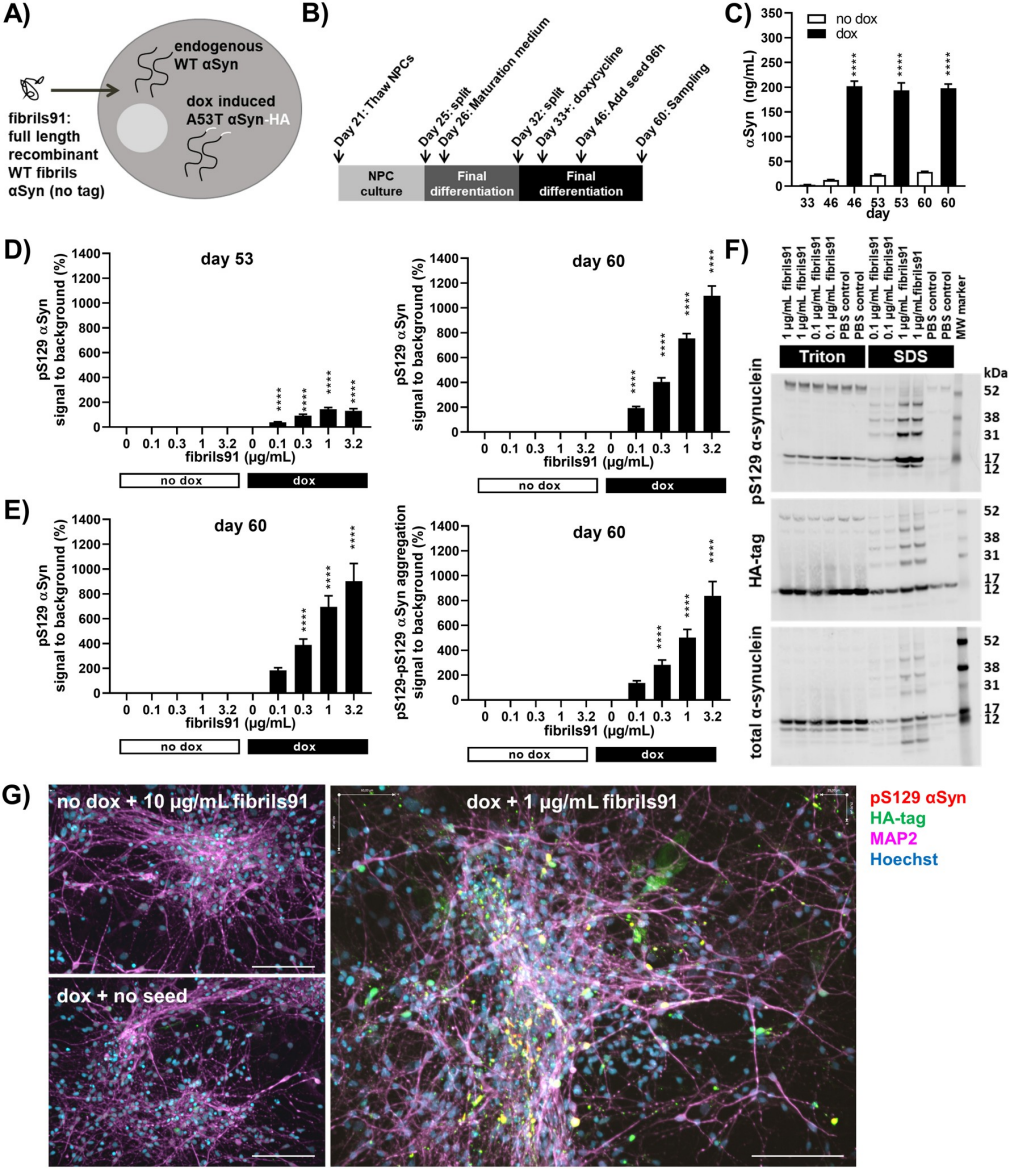
Exogenous fibrils91 seed α-synuclein aggregation in iPSC neurons expressing A53T α-synuclein. All graphs are shown as mean±SEM and ****p<0.0001. (A) Overview of the *in vitro* seeding model. (B) Outline of seeding experiments. Day 21 neuronal progenitors were thawed, expanded and split before changing from neuronal expansion medium to neuronal maturation medium day 26. A final split was performed day 32 followed by addition of doxycycline (dox) day 33. Fibrils91 seeds were added day 46 and left on for 96h before the media was refreshed. Cells were harvested for analysis day 60 unless otherwise indicated. (C) Electrochemiluminescence immunoassays showed upregulation of endogenous α-synuclein (αSyn) levels during neuronal differentiation. Doxycycline treatment resulted in stable A53T α-synuclein overexpression. Data were analyzed by one-way ANOVA followed by Sidaks’s multiple comparison test comparing doxycycline treated with non-treated for each timepoint (n = 3, m = 4). (D) FRET assays for pS129 α-synuclein showed a fibrils91-concentration and time dependent increase in the signal in doxycycline treated cells. Data were analyzed by one-way ANOVA followed by Dunnett’s multiple comparison test comparing to fibrils91 untreated samples for doxycycline treated and non-treated, respectively (n = 4, m = 6). (E) FRET assays for pS129 α-synuclein and aggregated pS129-pS129 α-synuclein on identical sample sets showed strong correlation. Data were analyzed by one-way ANOVA followed by Dunnett’s multiple comparison test comparing to fibrils91 untreated samples for doxycycline treated and non-treated, respectively (n = 4, m = 6). (F) Western blotting for pS129 α-synuclein, HA-tag and total α-synuclein in the Triton-soluble (Triton bar) and Triton-insoluble SDS-soluble (SDS bar) fractions from doxycycline treated neurons exposed to PBS, 0.1 μg/mL or 1 μg/mL fibrils91. The blots revealed a fibrils91-concentration dependent increase in α-synuclein in the Triton-insoluble SDS-soluble fractions. Representative blots of three experiments. (G) Immunocytochemistry confirmed that seeding was in neurons. Representative images of neurons stained for pS129 α-synuclein, HA-tag, MAP2 and Hoechst. Scale bars represent 100 μm. Neurons treated with fibrils91, but not doxycycline, were negative for pS129 α-synuclein demonstrating that the pS129 antibody does not stain the seed material (upper left). Neurons treated with doxycycline, but not fibrils91, were negative for HA-tag as the antibody was titrated down to show areas of high protein density (lower left). Neurons treated with fibrils91 and doxycycline revealed pS129 α-synuclein and HA-tag positive puncta (right). A rare non-neuronal cell positive for HA-tag stain is visible in the image. No seeding was seen in non-neuronal cells. Representative images from one out of three experiments.

De novo generated recombinant full-length wild-type human α-synuclein fibrils91 were quality controlled and fragmented (S7 Fig) and added to human iPSC neurons expressing either endogenous wild-type α-synuclein (i.e. neurons not treated with doxycycline) or endogenous wild-type α-synuclein plus overexpressed HA-tagged human A53T α-synuclein (i.e. neurons treated with doxycycline) as illustrated in Fig 2A. The experimental paradigm was as outlined in Fig 2B unless otherwise indicated. The expression of endogenous wild-type α-synuclein increased from day 46 to day 60. Doxycycline treatment of the cells resulted in stable overexpression of HA-tagged human A53T α-synuclein reaching levels of 16-, 9- and 7 folds above endogenous wild type protein on day 46, 53 and 60, respectively (Fig 2C). Overexpression of HA-tagged human A53T α-synuclein without fibrils91 treatment did not result in aggregation detectable by the pS129 α-synuclein FRET assay, the pS129-pS129 α-synuclein aggregation FRET assay, immunocytochemistry or fractionation (Fig 2D, 2E and 2G; S1 Raw images).

Exposing the doxycycline treated neurons to fibrils91 and harvesting them 1 week (day 53) and 2 weeks (day 60) after seed addition resulted in a fibrils91-concentration and time dependent increase in the FRET signal in the pS129 α-synuclein assay (Fig 2D). Analysing the samples in the pS129 α-synuclein assay as well as the pS129-pS129 α-synuclein aggregation assay resulted in similar curves indicating that the two assays are interchangeable (Fig 2E). Seeding was only observed in doxycycline treated cells demonstrating that the exogenous fibrils91 did not contributing to the signal in the assay, and that the endogenous wild-type α-synuclein alone was not enough to drive the templated aggregation in the cells.

To confirm the presence of seeded α-synuclein aggregation with an alternative method, we performed fractionation by ultra-centrifugation separating the cell lysates of neurons exposed to PBS, 0.1 μg/mL fibrils91 or 1 μg/mL fibrils91 into Triton-soluble and Triton-insoluble SDS-soluble fractions. Western blots for total α-synuclein and pS129 α-synuclein in the Triton-soluble fraction from doxycycline treated neurons revealed a 14 kDa and a 16 kDa band corresponding to full length wild-type endogenous and HA-tagged A53T α-synuclein, respectively (Fig 2F and S1 Raw images). Western blotting for pS129 α-synuclein in doxycycline treated neurons showed a fibrils91-concentration dependent increase in the Triton-insoluble SDS-soluble fractions (Fig 2F and S1 Raw images), which was absent in non-doxycycline treated neurons (S1 Raw images). This showed that the increase was not due to phosphorylation of the fibrils91 or formation of phosphorylated endogenous wild-type α-synuclein aggregates. Aggregation of HA-tagged A53T α-synuclein was confirmed by HA-tag blotting showing a fibrils91 concentration dependent increase in HA-band intensities in the Triton-insoluble SDS-soluble fraction in doxycycline treated neurons (Fig 2F and S1 Raw images). Western blots for total α-synuclein in the Triton-insoluble SDS-soluble fraction from doxycycline treated neurons further confirmed a fibrils91 concentration dependent increase in α-synuclein intensities (S1 Raw images), although some of signal comes from the fibrils91 as evident in the Triton-insoluble SDS-soluble fraction from fibrils91 exposed non-doxycycline treated neurons (S1 Raw images).

Based on the data from the fractionation and FRET-based α-synuclein assays, pS129 α-synuclein can be used as a proxy marker for α-synuclein aggregation. Immunocytochemistry for this epitope was therefore used to confirm that the templated aggregation was in neurons (Fig 2G). In line with the data from the fractionation and FRET-based α-synuclein assays, no pS129 α-synuclein signal was detectable in non-doxycycline treated neurons or neurons not exposed to fibrils91.

### A53T α-synuclein seeding can be modulated

**Fig 3:**
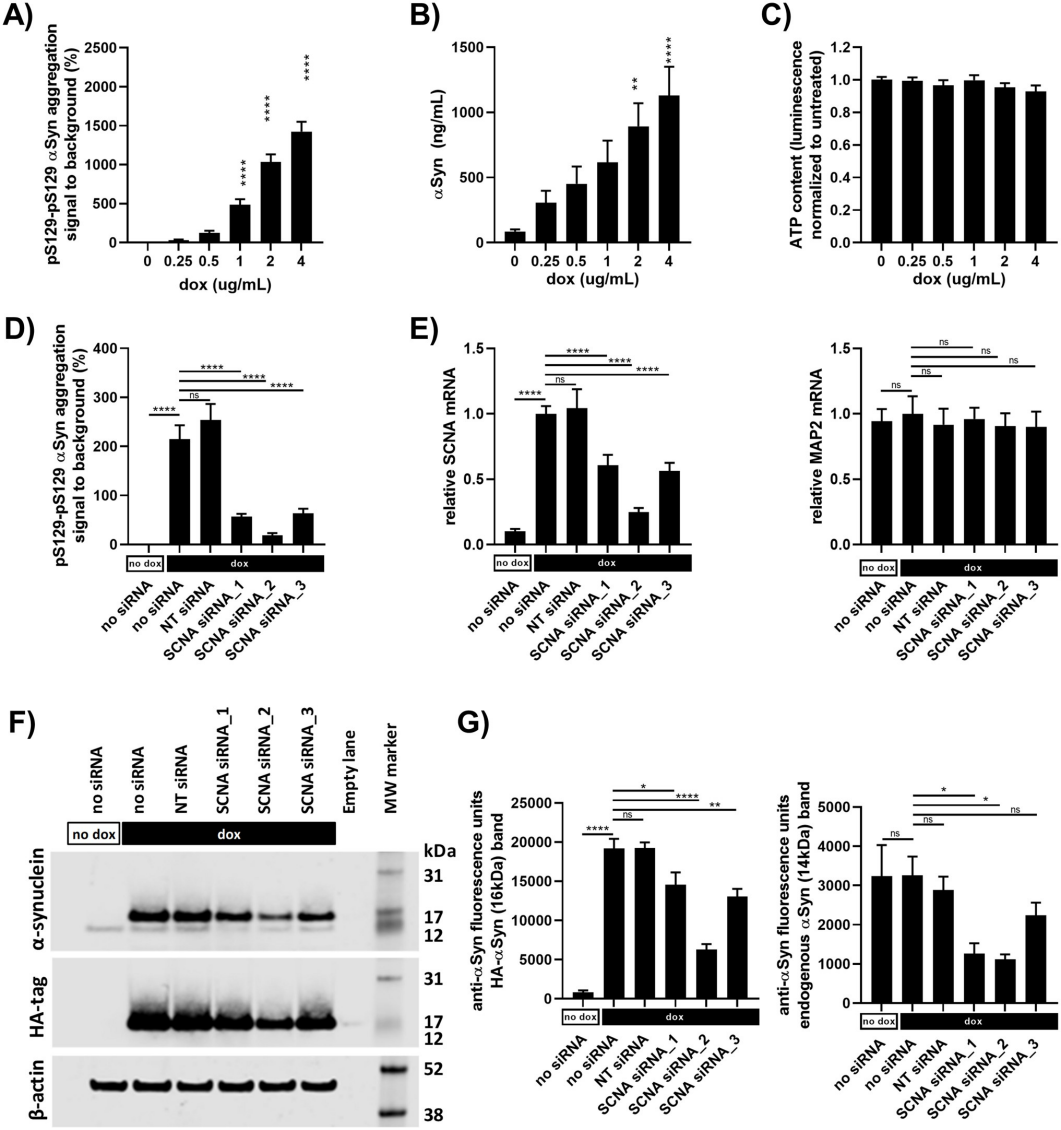
A53T α-synuclein seeding can be modulated. All graphs are shown as mean±SEM, ns = non-significant, *p<0.05, **p<0.01 and ****p<0.0001. (A) All samples were seeded with 0.1 μg/mL fibrils91. FRET assays for pS129-pS129 α-synuclein aggregation showed a doxycycline-concentration dependent increase in the signal. (B) Electrochemiluminescence immunoassay showed a doxycycline-concentration dependent increase in A53T α-synuclein expression. (C) Measuring the cellular ATP content revealed no cytotoxicity from addition of 0.25–4 μg/mL doxycycline. (D) All samples were seeded with 0.1 μg/mL fibrils91. FRET assays for pS129-pS129 α-synuclein aggregation showed a doxycycline dependent increase in the signal, which was reduced by addition of SCNA siRNAs, but not a pool of four non-targeting (NT) negative control siRNAs. (E) Relative gene expression analysis confirmed knockdown of SCNA mRNA by the SCNA siRNAs but not the NT siRNAs. No MAP2 off-targets effects were seen. (F) Western blotting for α-synuclein and HA-tag. Representative blots of two experiments. (G) Quantification of α-synuclein blotting in F) confirmed a doxycycline dependent increase α-synuclein expression. Both wild-type endogenous α-synuclein (14 kDa) and HA-tagged A53T α-synuclein (16 kDa) were reduced by addition of SCNA siRNAs but not a pool of four non-targeting (NT) negative control siRNAs. Data in A), B) and C) were analyzed by one-way ANOVA followed by Dunnett’s multiple comparison test comparing to doxycycline untreated samples (n = 3, m = 6). Data in D), E) and G) were analyzed by one-way ANOVA followed by Sidaks’s multiple comparison test comparing the groups indicated with horizontal black bars (D. n = 3, m = 6; E. n = 3, m = 4; G. n = 2, m = 2).

We aimed at generating an iPSC model of α-synuclein aggregation suitable for target validation and screening for modulators of α-synuclein aggregation. It was therefore crucial to demonstrate that the model has a suitable dynamic range and that gene modulation is feasible. First, α-synuclein aggregation was shown to increase with increasing concentration of doxycycline (Fig 3A) e.g. increasing A53T α-synuclein expression (Fig 3B), without measurable effect on cell survival (Fig 3C). This demonstrates that our standard experimental paradigm, using 1 μg/mL doxycycline, allows for both detection of increased and decreased aggregation.

To address the feasibility of gene modulation, we showed that pS129 α-synuclein aggregation could be reduced by addition of three different SCNA siRNAs but not by non-targeting siRNAs (Fig 3D). RT-qPCR gene expression analysis confirmed knockdown of SCNA mRNA (Fig 3E) and western blotting confirmed reduction of α-synuclein expression by SCNA siRNAs and no effect of non-targeting siRNAs (Fig 3F and 3G and S1 Raw images). There was correlation between α-synuclein mRNA knockdown, α-synuclein protein knockdown and α-synuclein aggregation (Fig 3D, 3E and 3G).

### Enzymes of the ubiquitination system affect A53T α-synuclein aggregation

**Fig 4:**
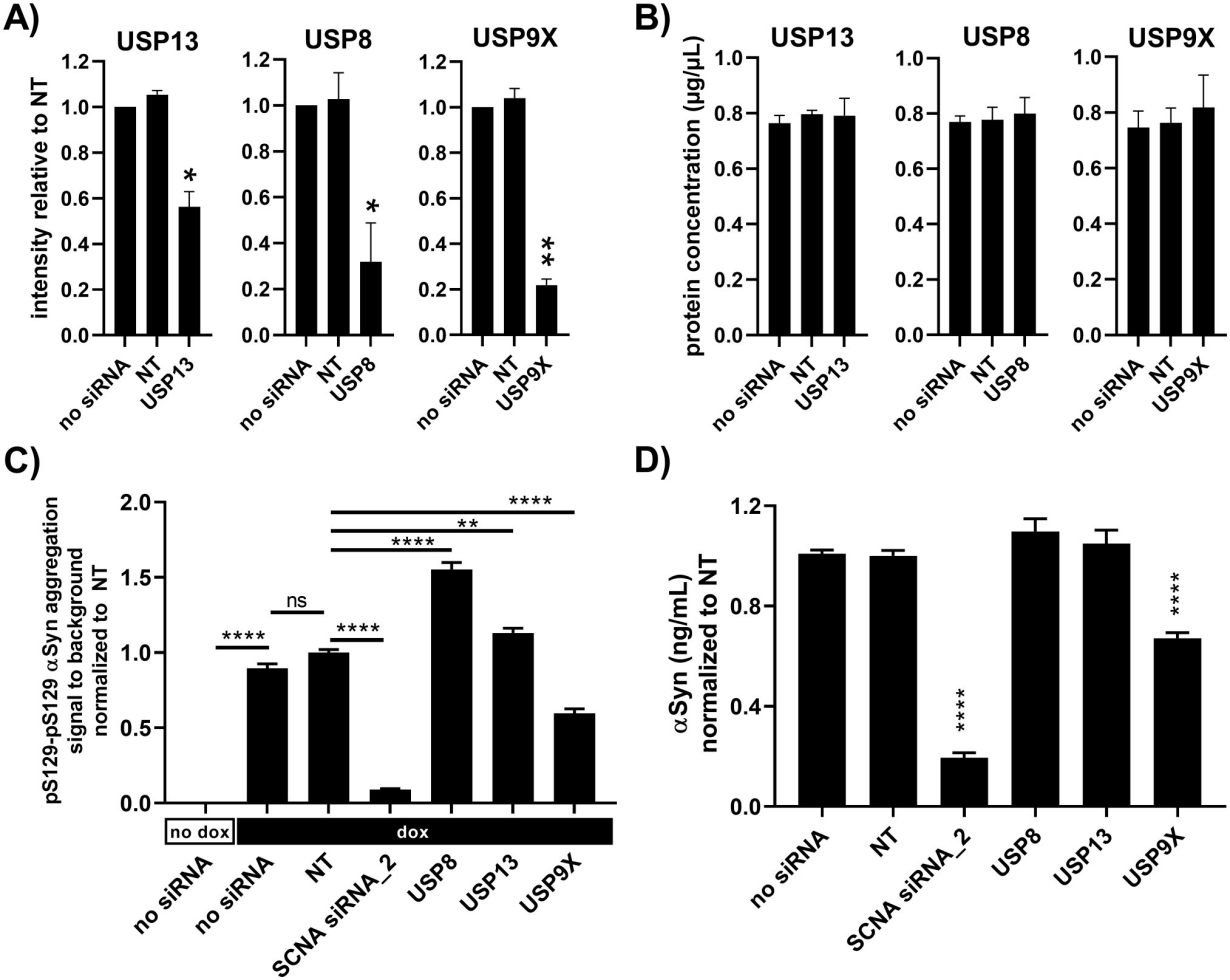
Enzymes of the ubiquitin system affect A53T α-synuclein aggregation. (A) Quantification of western blots (shown in S1 Raw images) showed reduction of USP13, USP8 and USP9X upon addition of specific pools of four siRNAs. The non-targeting (NT) negative control siRNAs were also pools of four siRNAs. (B) Quantification of total protein content in western blot samples showed no significant differences between treatments. (C) All samples were seeded with 0.1 μg/mL fibrils91. FRET assays for pS129-pS129 α-synuclein aggregation showed increased aggregation in response to USP8 and USP13 siRNAs and decreased aggregation in response to USP9X siRNAs. SCNA siRNAs was included as positive control and non-targeting (NT) siRNAs as negative control. (D) Electrochemiluminescence immunoassay showed down-regulation of α-synuclein protein levels in doxycycline treated neurons in response to SCNA and USP9X siRNAs. Data are shown as mean±SEM. Data in (A) and (B) were analyzed by one-way ANOVA followed by Dunnett’s multiple comparison test (n = 2, m = 2). Data in (C) and (D) were analyzed by one-way ANOVA followed by Sidaks’s multiple comparison test (n = 3, m = 6). ns = non-significant, *p<0.05 and **p<0.01 and ****p<0.0001.

USP8, USP13 and USP9X are all enzymes of the ubiquitination system that are reported to modulate α-synuclein expression or aggregation [19–21]. These genes were therefore knocked down using pools of four siRNAs for each gene to investigate if our model could capture previous findings. Quantifications of western blots for the enzymes showed statistically significant reduction of USP13, USP8 and USP9X protein levels upon siRNA treatment (Fig 4A and S1 Raw images). None of the siRNAs induced cytotoxicity as determined by total protein content determinations (Fig 4B). USP13 and USP8 knockdown led to increased α-synuclein aggregation (Fig 4C) without affecting α-synuclein protein levels (Fig 4D). USP9X knockdown reduced α-synuclein aggregation and protein levels (Fig 4C and 4D).

## Discussion

We aimed at generating an iPSC model to investigate α-synuclein aggregation in a robust and reproducible system with a good throughput. The first step to reach that goal was to generate the gene-editing iPSC BIONi010-C-24 line with doxycycline inducible HA-tagged α-synuclein A53T expression. Differentiation of 5 million iPSCs resulted in 170±70 million neuronal progenitors, which could be cryopreserved. Thawing and further differentiation provided enough cells for seeding 10,200 x 96-wells (50.000 cells/well day 32), demonstrating the feasibility of upscaling the iPSC neuron production for drug discovery.

Three neuronal progenitor batches were generated, which could be matured to neurons with high reproducibility. Upon maturation, the majority of the cells were of neuronal identity exhibiting clear neuronal morphology and expression at protein and mRNA levels of several pan-neuronal markers. Although the expressed tau isoform indicates embryonic identity, these postmitotic neurons responded to depolarization of the plasma membrane, expressed functional neurotransmitter receptors and showed robust spontaneous calcium oscillations indicative of their functional maturity. Overall, the human identity and the reproducible neuronal differentiation qualify the model for use in pharmaceutical neuroscience.

Overexpression of HA-tagged human A53T α-synuclein without fibrils91 treatment did not result in detectable aggregation in our model although reported by others [6–9]. The reasons for the difference could be many; the use of a patient genetic background, the detection assays used, antibody specificity, the seed material, the culture conditions, etc.

Exposing the doxycycline treated neurons to recombinant human wild-type fibrils91 resulted in increased FRET signals in pS129 α-synuclein and pS129-pS129 α-synuclein aggregation assays, increased numbers of pS129 α-synuclein positive puncta in immunocytochemistry and the appearance of Triton-insoluble SDS-soluble HA-tagged α-synuclein. The α-synuclein aggregation FRET-based assay provides a high throughput endpoint addressing aggregation directly, which is preferable in the search for therapeutical approaches modulating this feature of Parkinson’s Disease. Seeding of endogenous wild-type α-synuclein was not detectable in our system, although reported previously in studies using iPSC derived neurons [10, 11]. Absence of endogenous seeding can be considered an advantage in a drug discovery setting as it provides a control, demonstrating that the fibrils91 themselves are not detected by the assays.

Our standard experimental paradigm, using 1 μg/mL doxycycline, allowed for both detection of increased and decrease aggregation as demonstrated by the dependency of α-synuclein aggregation on intracellular A53T α-synuclein levels. Furthermore, the fibrils91 induced seeding could be opposed using SNCA siRNAs. This confirms that gene modulation is achievable in the model, and that it has a suitable dynamic range to detect both increased and decreased aggregation, making it suitable to be used for genetic screening for aggregation modulators.

The USP8, USP13 and USP9X enzymes of the ubiquitination system have been reported by others to modulate α-synuclein expression or aggregation [19–21]. Liu *et al*. describe increased α-synuclein ubiquitination and clearance upon USP13 shRNA injection in substantia nigra of mice [20]. In contrast, we found no effect of USP13 knockdown on monomeric α-synuclein protein levels. Liu *et al*. also report that USP13 knockdown protects from cell death induced by lentiviral α-synuclein overexpression *in vivo*. In contrast, we find increased formation of aggregation upon seeding pointing to a non-protective effect of USP13 knockdown. USP9X knockdown in SH-SY5Y cells is described to promote the formation of monoubiquitinated α-synuclein species, thus increasing the steady state levels of total α-synuclein and enhancing the formation of toxic α-synuclein inclusions upon proteolytic inhibition [21]. Knockdown of USP9X in our model based on iPSC neurons resulted in decreased monomeric α-synuclein levels and reduced seeded aggregation. USP8 knockdown in SH-SY5Y cells reduced α-synuclein levels and protected against α-synuclein expression induced toxicity (rough eye phenotype) in Drosophila flies [19]. In our iPSC neuron model, USP8 knockdown did not affect monomeric α-synuclein levels while increasing seeded aggregation. The discrepancies between these findings may be due to factors such as the animal species, α-synuclein species, *in vivo* versus *in vitro* system, the cell type, the protein expression levels, off-target effects of siRNAs/shRNAs, etc. This strongly highlights the importance of confirming biological findings in independent model systems. In short, our data indicate that USP8 and USP13 facilitate degradation of aggregated α-synuclein in seeded neurons without affecting levels of monomeric α-synuclein (data from neurons not exposed to fibrils).

We succeed in generating a reproducible assay with a robust seeding window suitable for genetic screens. The cortical differentiation protocol was selected due to its high reproducibility, HA-tagged α-synuclein was used to be able to differentiate endogenously formed fibrils and A53T α-synuclein was used to increase the assay window as it is more aggregation prone than wildtype α-synuclein [18]. It remains to be shown whether the same assay build on dopaminergic neurons and non-tagged wildtype α-synuclein would give identical results to understand better translatability into humans and the broader Parkinson’s patient cohort.

In overall conclusion, our study shows that doxycycline induced HA-tagged A53T α-synuclein expression can be used to generate a robust humanized seeding model. The model exhibits two fundamental characteristics for genetic screens and target validation in pharmacological research: a high reproducibility and a remarkable versatility for throughput studies.

## Supporting information

S1 Raw images Original uncropped images for western blots. (PDF)

S1 Fig Quality control of BIONi010-C-24 iPSCs (A) Purity was confirmed by three PCRs addressing the integration of Neo and Puro as well as the absence of AAVS1 wild-type sequence: The Neo integration PCR (left) detects the correct insertion of the construct containing the reverse tetracycline transactivator (M2rtTA). The Puro integration PCR (middle) detects the insertion of the construct with the SNCA cDNA. The absence of the wild-type sequence of the AAVS1 locus (right) confirms that both alleles of the AAVS1 locus contain an integration and that no contaminating wild-type cells are present. X indicate samples that are not part of this study. (B) Normal karyotype was shown by G-banding: The iPSCs were incubated with Colcemid (Gibco) for 1.5 h and split with Accutase. Next, cells were incubated with 0.075M KCl for 30 minutes at 37° C and afterwards fixed with a mixture of 25% acidic acid and 75% methanol. Fixed cells were placed at -20°C overnight and then shipped for G-band karyotyping at the Institute of Medical Genetics and Applied Genomics, University of Tübingen. A normal male karyotype was found, 46,XY (C) Immunocytochemistry demonstrated expression of the pluripotency markers Oct-4, Sox2 and Tra-1-60: The cells were fixed with ice cold methanol, blocked and permeabilized with PBS containing 2% BSA and 0.1% Triton-X-100, and incubated with the primary antibodies over night at 4° C (anti-Oct4, polyclonal goat, Abcam ab27985, RRID:AB_776898, 1:100; anti-Sox2, polyclonal rabbit, Abcam ab97959, RRID:AB_2341193, 1:100; anti-Tra-1-60, monoclonal mouse, Merck MAB4360, RRID:AB_2119183, 1:100). The cells were washed and incubated with the secondary antibody and Hoechst for 1 hour (donkey anti-goat Alexa fluor 488, Invitrogen A11055 RRID: AB_2534102, 1:200; donkey anti-mouse Alexa fluor 488, Life technologies A21202 RRID: AB_141607, 1:200; donkey anti-rabbit Alexa fluor 488, Life technologies A21206 RRID: AB_2535792, 1:200). The cells were loaded with mounting solution containing DAPI from Invitrogen and investigated by fluorescence microscopy. (D) Pluripotency was supported by the capability of trilineage differentiation *in vitro*, as confirmed by flow cytometry showing expression of the ectodermal marker PAX6 (5662388 BD Bioscience, Cy5, 1:50) and Sox1 (561592 BD Bioscience, PE, 1:50), the mesodermal marker CD56 (555518 BD Bioscience, APC, 1:25) and CD34 (555822 BD Bioscience, PE, 1:25) and the endodermal marker Sox17 (562594 BD Bioscience, AF, 1:50) and CD184 (555974 BD Bioscience, PE, 1:25): The iPSCs were split with accutase into a well of a 12-well plate with stem cell medium on matrigel in different densities: 200,000 cells/cm2 for ecto- and endoderm and 50,000 cells/cm2 for mesoderm. For ectodermal differentiation, the medium was changed to neural induction medium (50% DMEM F12 and 50% Neurobasal medium, 1X B27 without retinoic acid, 1X N2 supplement, 1X glutamax, 1X Pen/Strep (all Gibco), 10 μM SB431542, 0.1 μM LDN193189 (both from Selleckchem)) on day one. The medium was changed every day until day six. For endodermal differentiation, the medium was changed to MCDB131-1 medium (MCDB131 medium, 1.5 g/L NaHCO3, 1X glutamax, 1X Pen/Strep (all Gibco), 10 mM glucose (Sigma), 0.5% BSA) on day one including 3 μM CHIR99021 (Selleckchem) and 100ng/mL Activin A (Cell Guidance Systems). On day two, CHIR99021 was withdrawn and MCDB131-1 medium with activin A was changed every day until day six. For mesodermal differentiation, the medium was replaced by mesodermal induction medium (APEL medium (Gibco), 25 μg/mL Activin A (Cell Guidance Systems), 30 ng/mL BMP4 (Peprotech), 50 ng/mL VEGF (peprotech), 1.5 μM CHIR99021 (Selleckchem)), which was left on the cells for two days. On day three, the medium was changed to vascular specification medium (APEL medium, 50 ng/mL VEGF, 10 μM SB431542 (Selleckchem)), which was then changed every day until day six. The Staining Buffer Set from Invitrogen was used for flow cytometry analysis. iPSCs were detached with Accutase for 5–10 minutes and 200,000 cells were fixed in 0.5 mL of the fixation/permeabilization buffer according to the guidelines. After 30 minutes incubation at RT, the cells were washed three times with permeabilization buffer and then incubated for 45 minutes with 100 μL of antibody. Cells were washed additionally three times and afterwards run at a calibrated BD Accuri C6 flow cytometer (analyze 50.000 cells at high speed in 150 μl buffer).
(TIF)

S2 Fig Immunocytochemistry analysis on day 0, 12 and 25 of differentiation. Immunocytochemistry showed expression of Oct4 protein at day 0, Oct-4 was downregulated upon neuronal induction, while the neuronal progenitor marker nestin was up-regulated. Pax6, a marker of dorsal forebrain regionalization, showed transient protein expression. At all three timepoints, the majority of cells were positive for the proliferation marker Ki67. The number of cells positive for the neuronal cytoskeleton markers tau and βIII-tubulin (βIII-tub) increase with time of differentiation. Scale bars. 200 μm. Representative images from one out of three independent neuronal differentiations.
(TIF)

S3 Fig Immunocytochemistry analysis on day 45 and 60 of differentiation. Immunocytochemistry of iPSC neurons at day 45 and 60 of differentiation showing expression of neuronal cytoskeleton markers tau, doublecortin (DCX) and βIII-tubulin (βIII-tub), the neurotransmitter GABA and the synaptic protein synapsin-I (syn-I) in most cells. On day 45 and 60, only small populations were positive for the neuroprogenitor markers nestin and the proliferation marker Ki67. Scale bars are 200 μm. Three of the images (marked with a star) are identical to those in Fig 1C. Representative images from one out of three independent neuronal differentiations.
(TIF)

S4 Fig Immunocytochemistry analysis on day 60 of differentiation. A GFAP positive subpopulation could be identified day 60. Scale bars are 200 μm. Representative images from one out of three independent neuronal differentiations.
(TIF)

S5 Fig Neurotransmitter induced changes in intracellular calcium in neurons from three independent neuronal differentiations. Changes in intracellular calcium in day 52–53 neurons following application of buffer control and the neurotransmitters, glutamate (300 μM glutamate +10 μM glycine), NMDA (40 μM) and GABA (100 μM), or depolarization of the membrane potential by addition of extracellular potassium chloride (25 mM). Four of the graphs (marked with a star) are identical to those in Fig 1F. The three neuronal differentiations (diff. 1, 2 and 3) are independent.
(TIF)

S6 Fig Spontaneous calcium oscillations in neurons from three independent neuronal differentiations. Spontaneous oscillations in day 52/53 and 59/60 neurons are eliminated by addition (timepoint marked with triangle) of 1 μM tetrodotoxin (grey traces) but not buffer (black traces). One of the graphs (marked with a star) is identical to those in Fig 1G. The three neuronal differentiations (diff. 1, 2 and 3) are independent.
(TIF)

S7 Fig Characterization of alpha-synuclein fibrils91. Transmission electron microscopy images before (A) and after fragmentation (B), scale bar: 200nm. (C) Proteinase K (PK, 3.8 μg/mL) degradation patterns of alpha-synuclein fibrils 91 (100μM monomer concentration) monitored over time on Coomassie stained SDS–PAGE (15%). Time (min) and molecular weight markers (MW, kDa) are shown on the top and left sides of the gels, respectively.
(TIF)
