# Supplementary material for: Establishment of a human induced pluripotent stem cell neuronal model for identification of modulators of A53T α-synuclein levels and aggregation
Source: PLoS One. 2021 Dec 21;16(12):e0261536. doi: 10.1371/journal.pone.0261536 (PMC8691628; doi:10.1371/journal.pone.0261536)

Fig 1B: Full blot of Fig 1B.

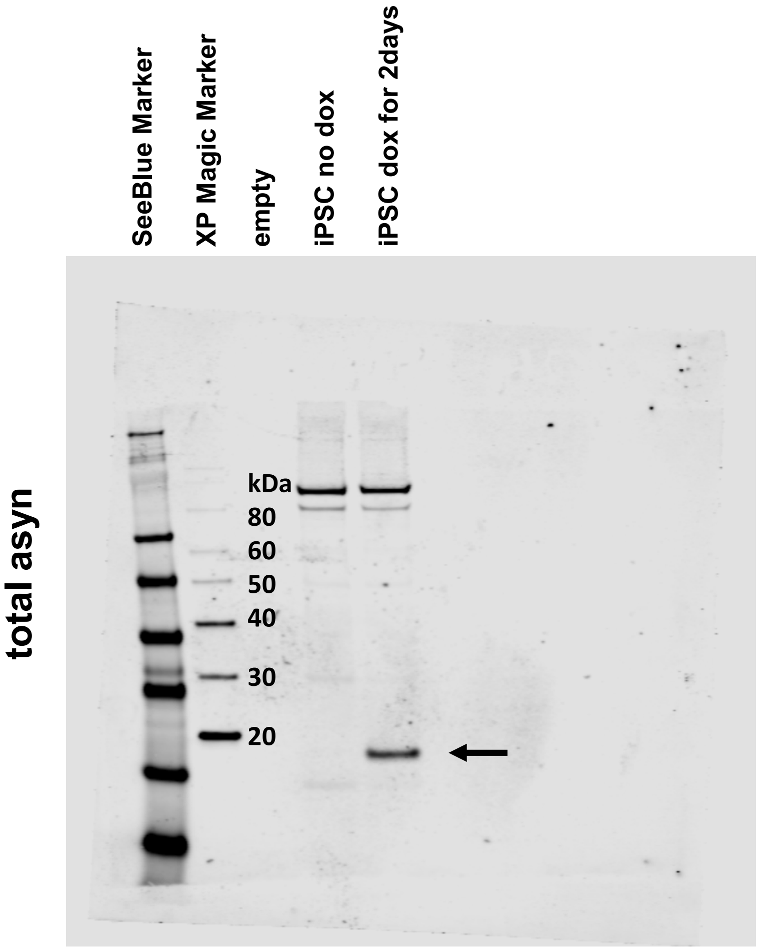

Fig 1D: Full blots of the representative neuronal differentiation shown in Fig 1D (marked with a star) and two additional neuronal differentiations. Day 0 is undifferentiated iPSCs and day 59 is iPSC neurons derived in three independent neuronal differentiations (diff. 1, 2 and 3). X indicate samples that are not part of this study.

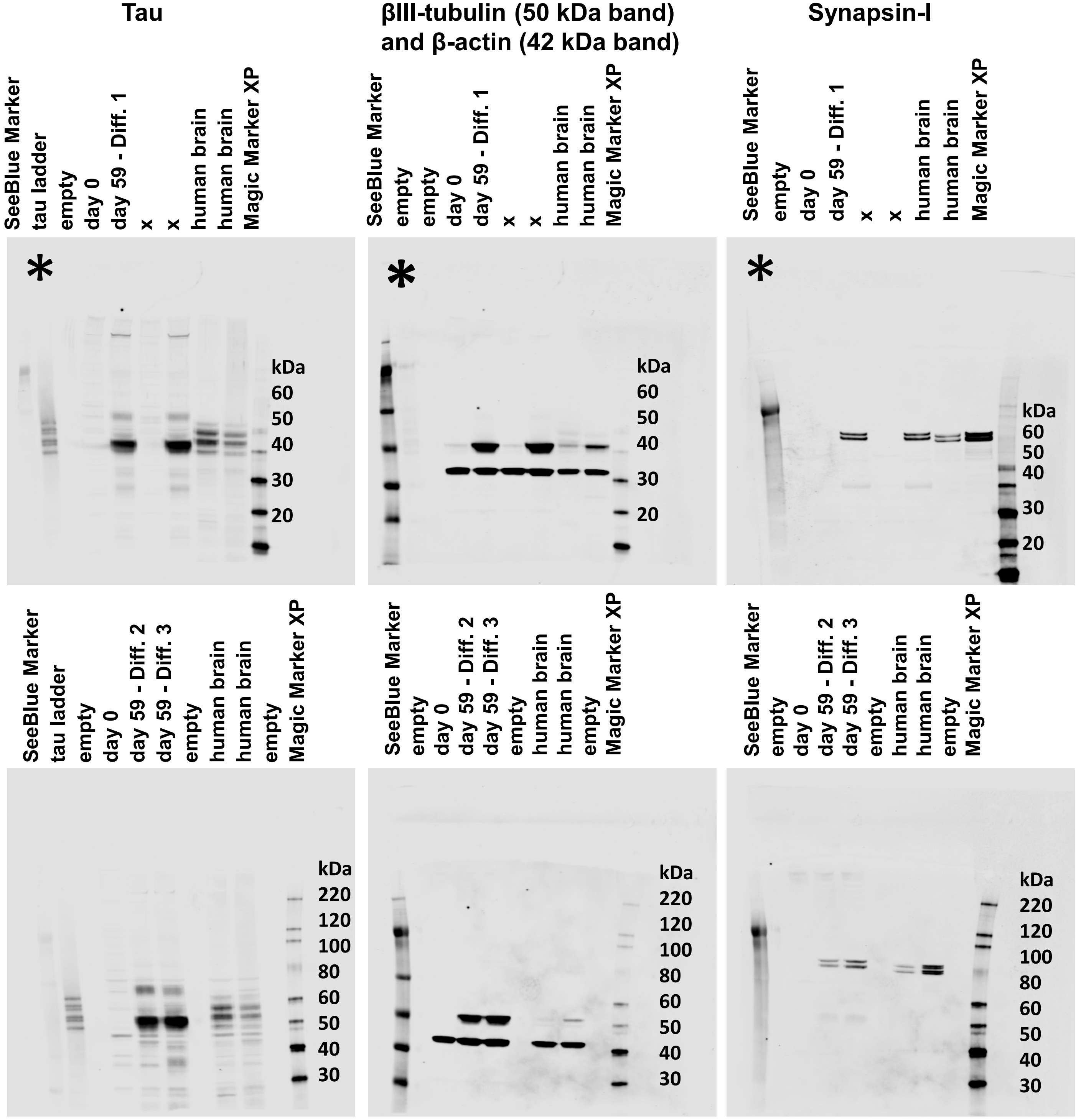

Fig 2F: Full blots of the representative experiment shown in Fig 2F (marked with a star) and two additional experiments. The experimental conditions in A) are as described in Fig 2F. The conditions in B)are identical, except that the neurons were not exposed to doxycycline.

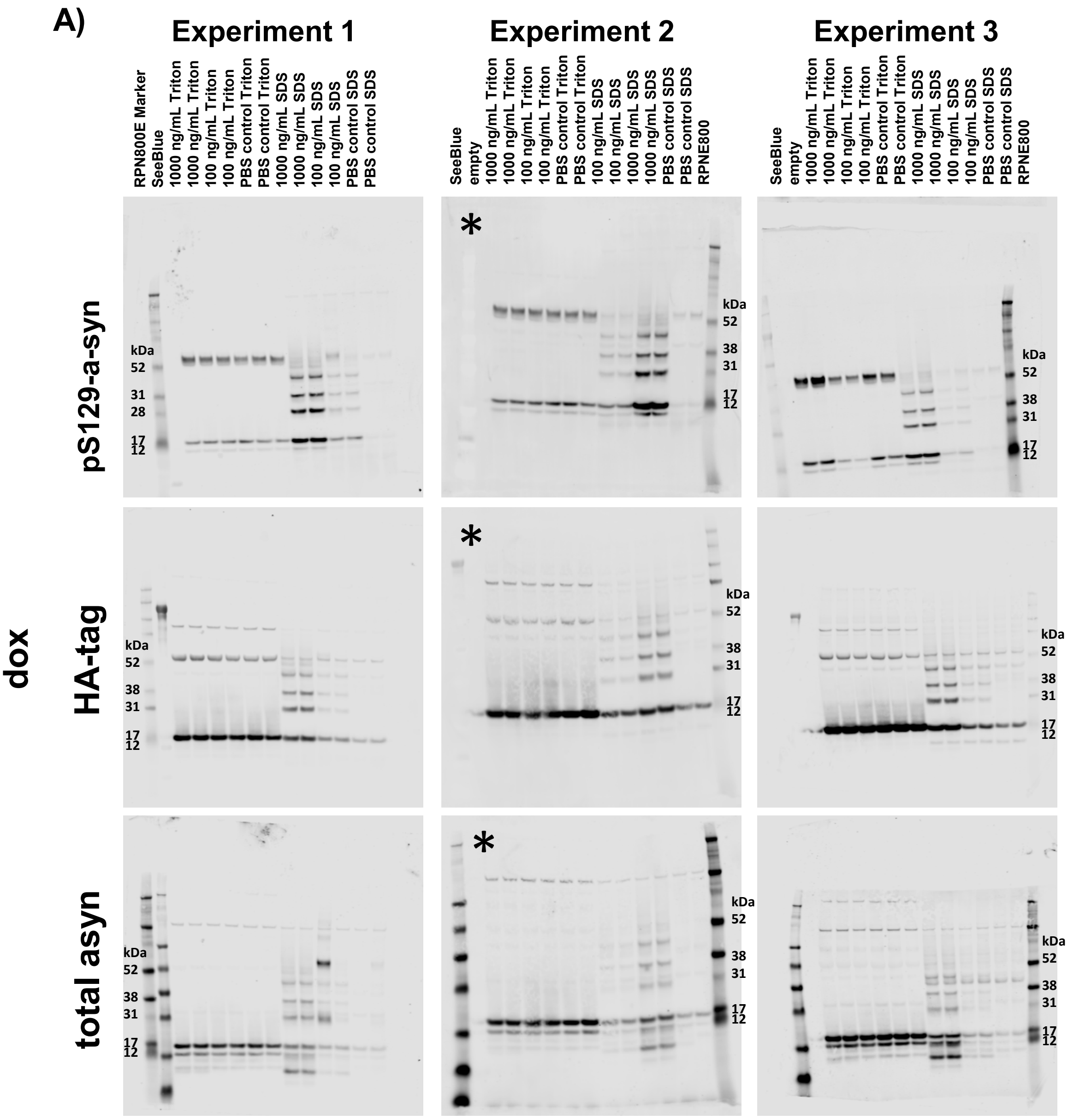

Fig 2F continued:

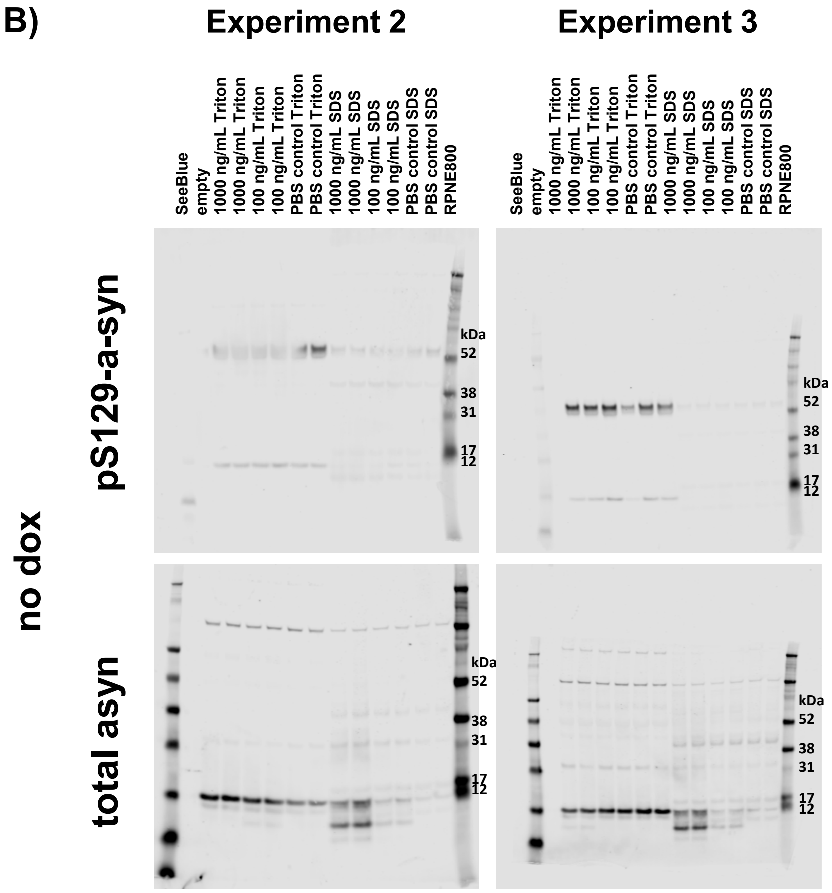

Fig 3F: Full blots of the representative experiment shown in Fig 3F (marked with a star) and one additional experiment.

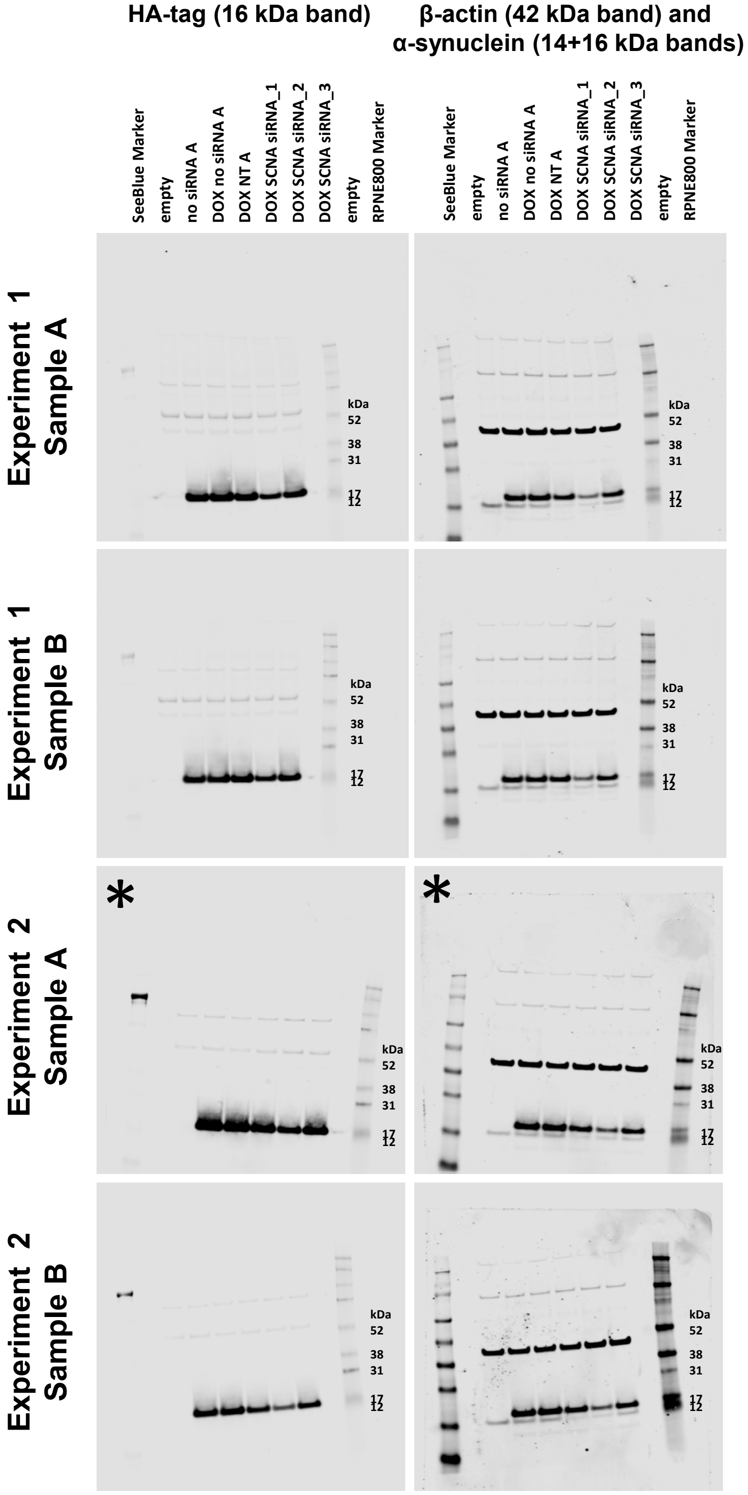

Fig 4A: Full blots of the experiment quantified in Fig 4A. The arrows indicate the USP13 (A), USP9X (B) and USP8 (C) bands. X indicate samples that are not part of this study.

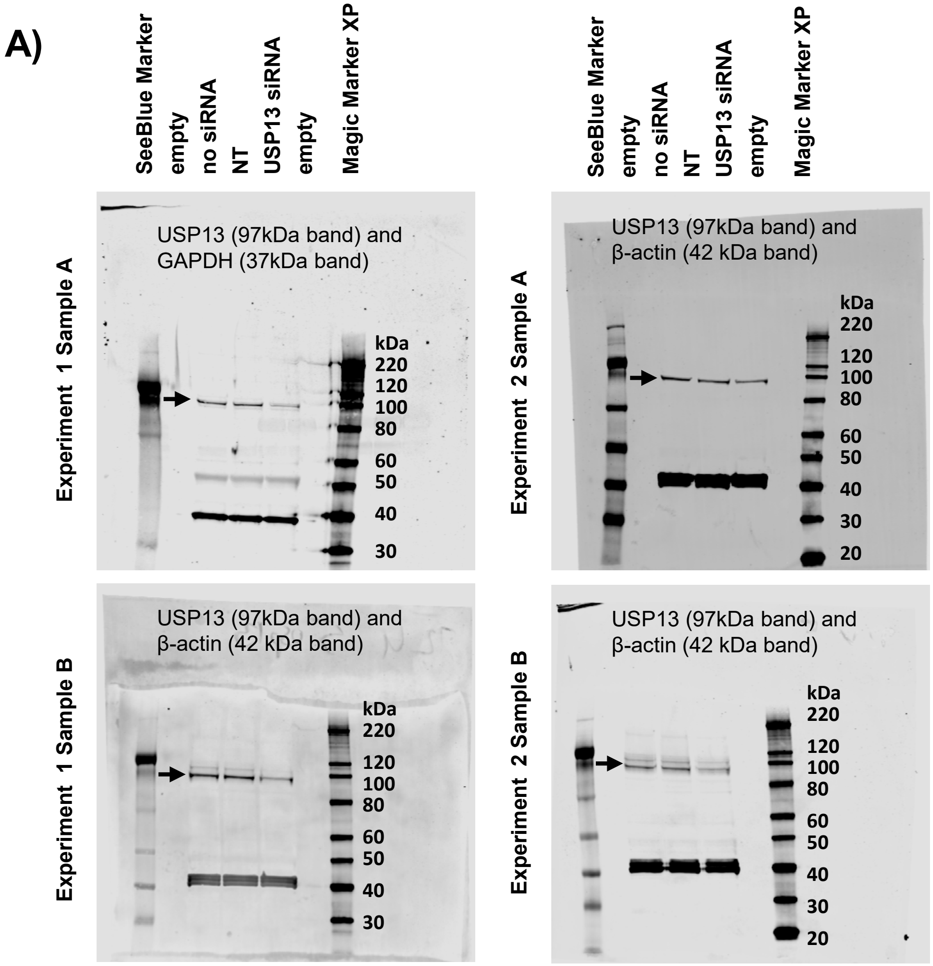

Fig 4A continued:

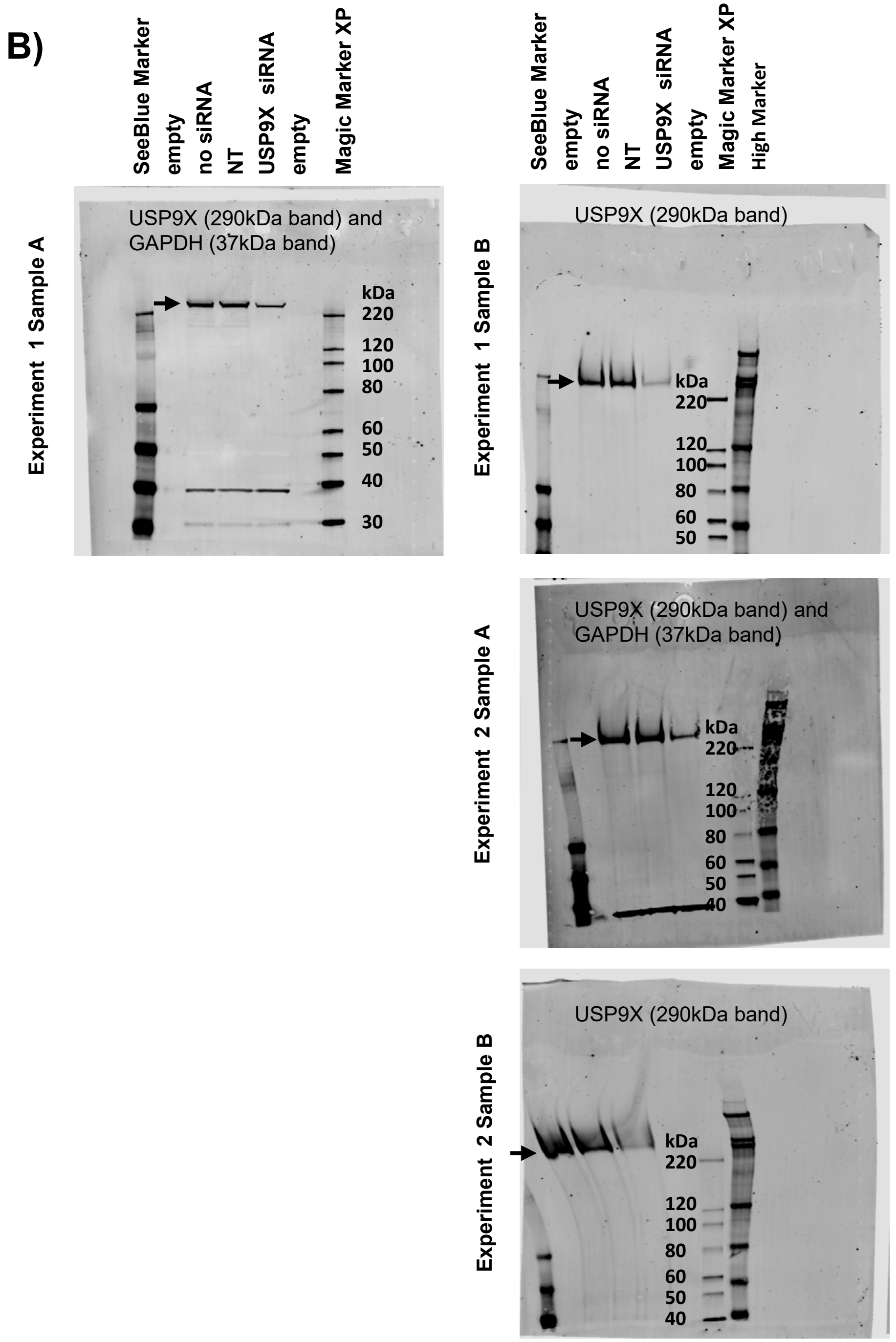

Fig 4A continued:

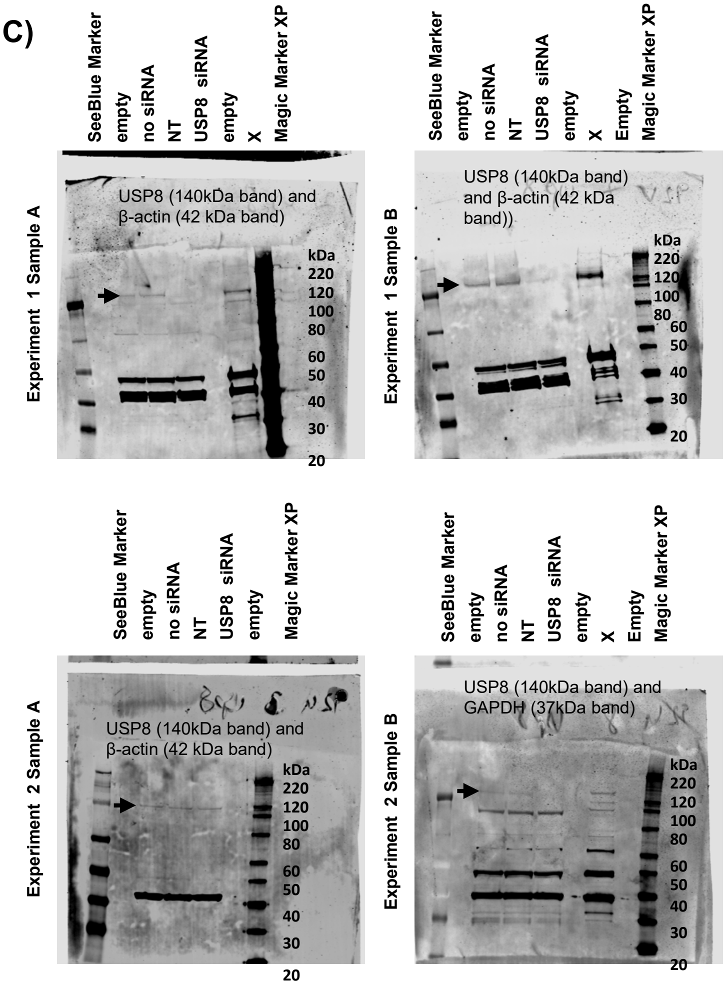

Supplement: S1 Raw images — (PDF) [file pone.0261536.s001.pdf]
