# Supplementary figures and images for: Establishment of a human induced pluripotent stem cell neuronal model for identification of modulators of A53T α-synuclein levels and aggregation
Source: PLoS One. 2021 Dec 21;16(12):e0261536. doi: 10.1371/journal.pone.0261536 (PMC8691628; doi:10.1371/journal.pone.0261536)

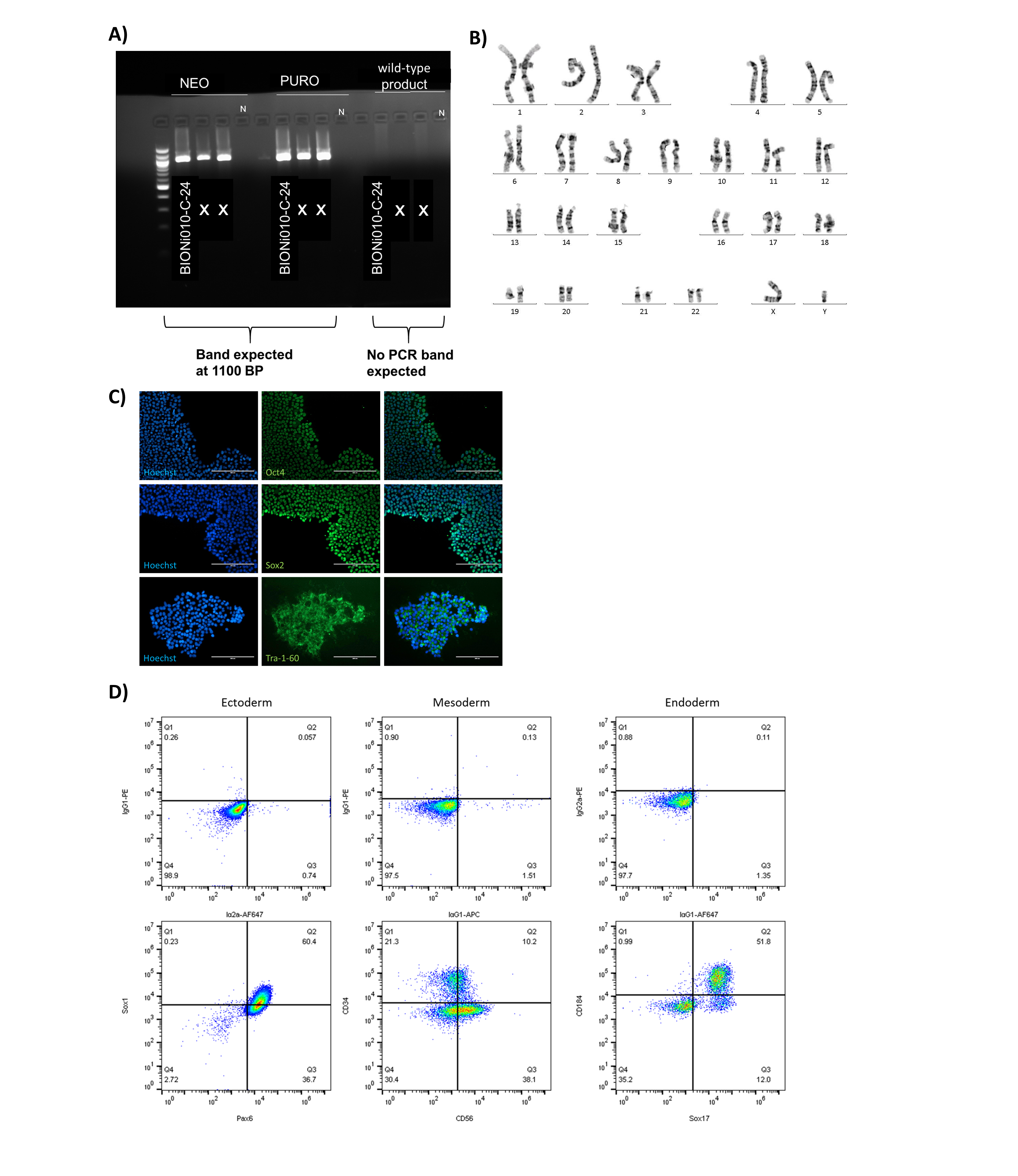

Supplement: S1 Fig — Quality control of BIONi010-C-24 iPSCs (A) Purity was confirmed by three PCRs addressing the integration of Neo and Puro as well as the absence of AAVS1 wild-type sequence: The Neo integration PCR (left) detects the correct insertion of the construct containing the reverse tetracycline transactivator (M2rtTA). The Puro integration PCR (middle) detects the insertion of the construct with the SNCA cDNA. The absence of the wild-type sequence of the AAVS1 locus (right) confirms that both alleles of the AAVS1 locus contain an integration and that no contaminating wild-type cells are present. X indicate samples that are not part of this study. (B) Normal karyotype was shown by G-banding: The iPSCs were incubated with Colcemid (Gibco) for 1.5 h and split with Accutase. Next, cells were incubated with 0.075M KCl for 30 minutes at 37° C and afterwards fixed with a mixture of 25% acidic acid and 75% methanol. Fixed cells were placed at -20°C overnight and then shipped for G-band karyotyping at the Institute of Medical Genetics and Applied Genomics, University of Tübingen. A normal male karyotype was found, 46,XY (C) Immunocytochemistry demonstrated expression of the pluripotency markers Oct-4, Sox2 and Tra-1-60: The cells were fixed with ice cold methanol, blocked and permeabilized with PBS containing 2% BSA and 0.1% Triton-X-100, and incubated with the primary antibodies over night at 4° C (anti-Oct4, polyclonal goat, Abcam ab27985, RRID:AB_776898, 1:100; anti-Sox2, polyclonal rabbit, Abcam ab97959, RRID:AB_2341193, 1:100; anti-Tra-1-60, monoclonal mouse, Merck MAB4360, RRID:AB_2119183, 1:100). The cells were washed and incubated with the secondary antibody and Hoechst for 1 hour (donkey anti-goat Alexa fluor 488, Invitrogen A11055 RRID: AB_2534102, 1:200; donkey anti-mouse Alexa fluor 488, Life technologies A21202 RRID: AB_141607, 1:200; donkey anti-rabbit Alexa fluor 488, Life technologies A21206 RRID: AB_2535792, 1:200). The cells were loaded with mounting solution contai [file pone.0261536.s002.tif]

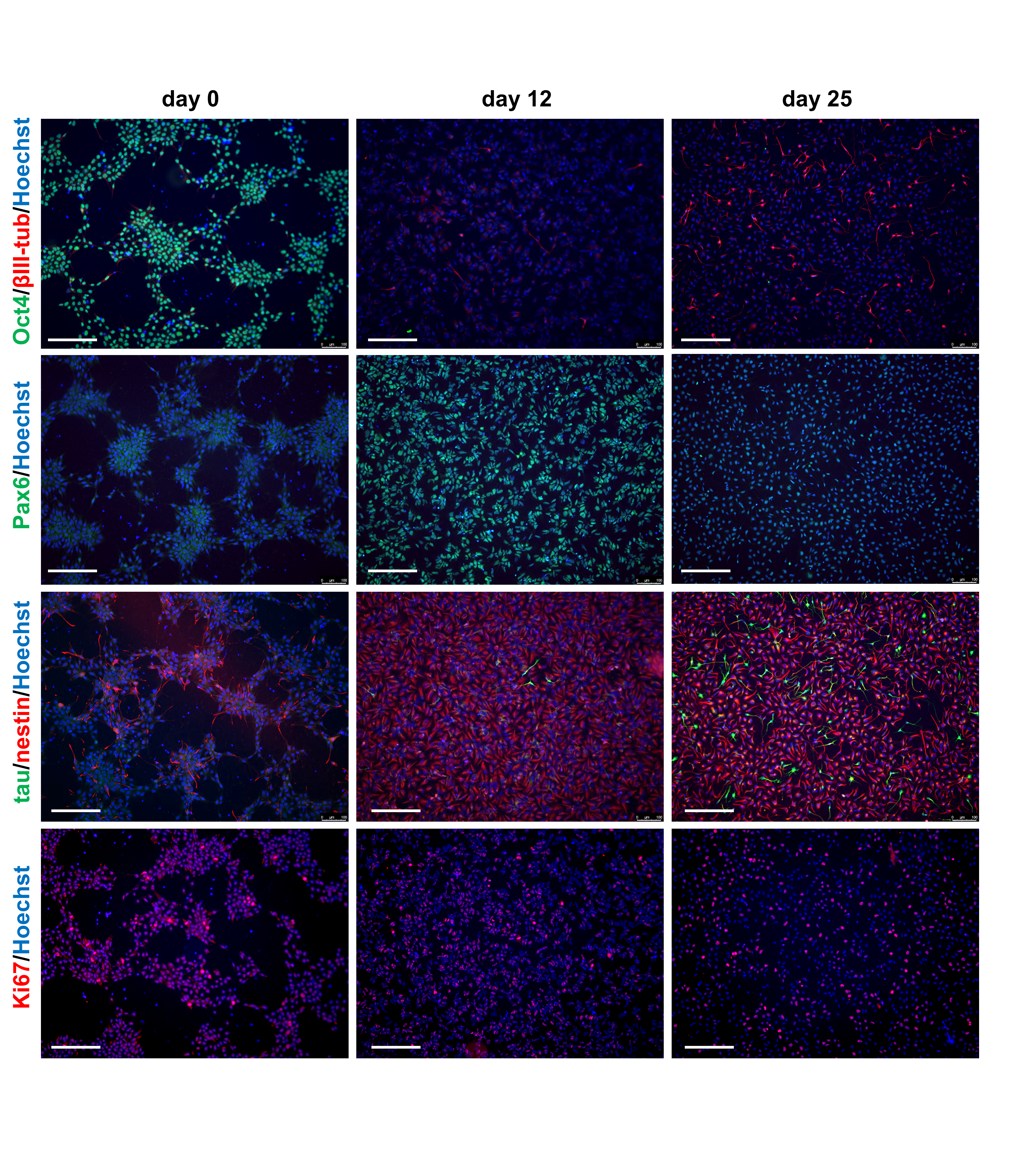

Supplement: S2 Fig — Immunocytochemistry showed expression of Oct4 protein at day 0, Oct-4 was downregulated upon neuronal induction, while the neuronal progenitor marker nestin was up-regulated. Pax6, a marker of dorsal forebrain regionalization, showed transient protein expression. At all three timepoints, the majority of cells were positive for the proliferation marker Ki67. The number of cells positive for the neuronal cytoskeleton markers tau and βIII-tubulin (βIII-tub) increase with time of differentiation. Scale bars. 200 μm. Representative images from one out of three independent neuronal differentiations. (TIF) [file pone.0261536.s003.tif]

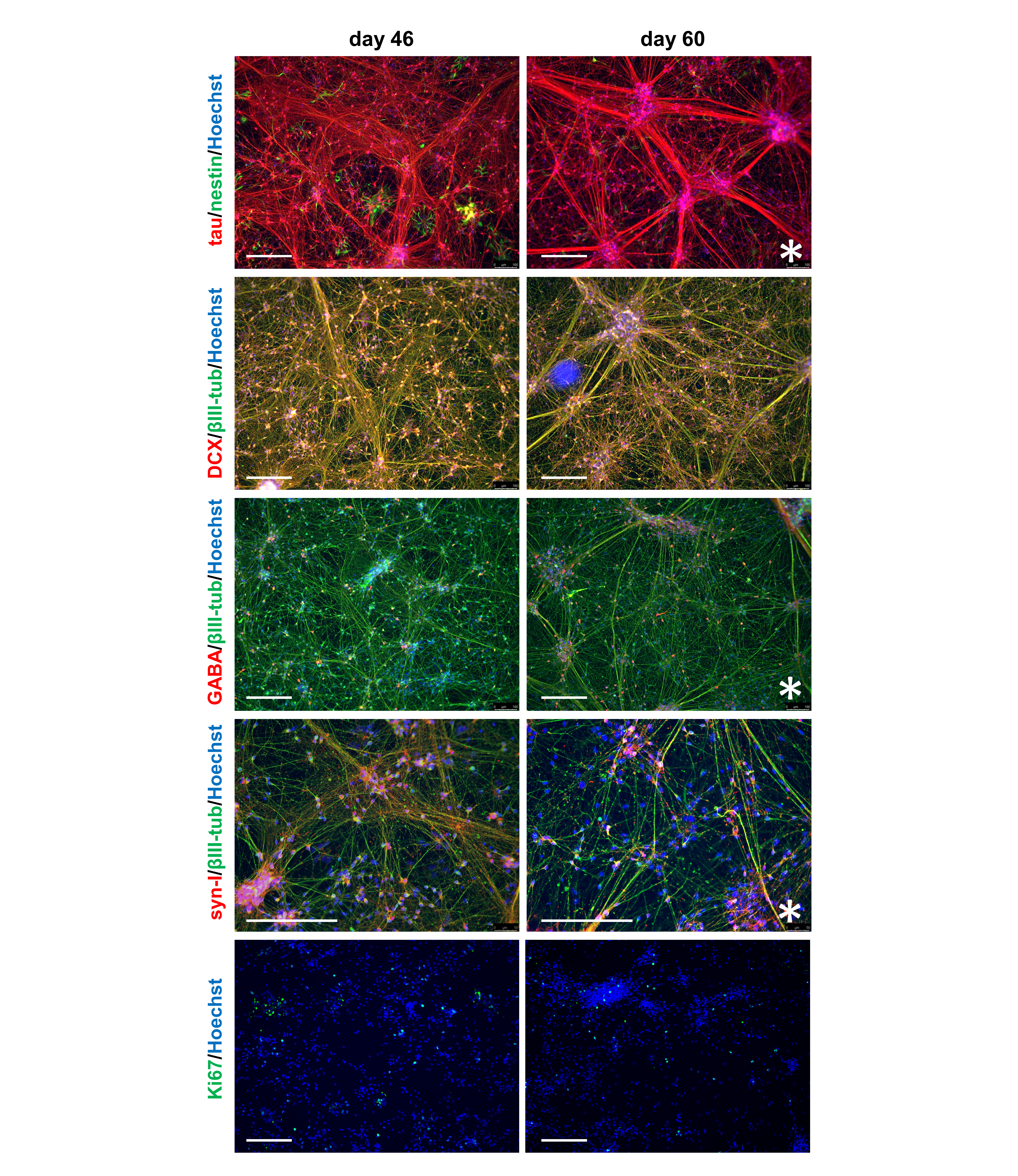

Supplement: S3 Fig — Immunocytochemistry of iPSC neurons at day 45 and 60 of differentiation showing expression of neuronal cytoskeleton markers tau, doublecortin (DCX) and βIII-tubulin (βIII-tub), the neurotransmitter GABA and the synaptic protein synapsin-I (syn-I) in most cells. On day 45 and 60, only small populations were positive for the neuroprogenitor markers nestin and the proliferation marker Ki67. Scale bars are 200 μm. Three of the images (marked with a star) are identical to those in Fig 1C. Representative images from one out of three independent neuronal differentiations. (TIF) [file pone.0261536.s004.tif]

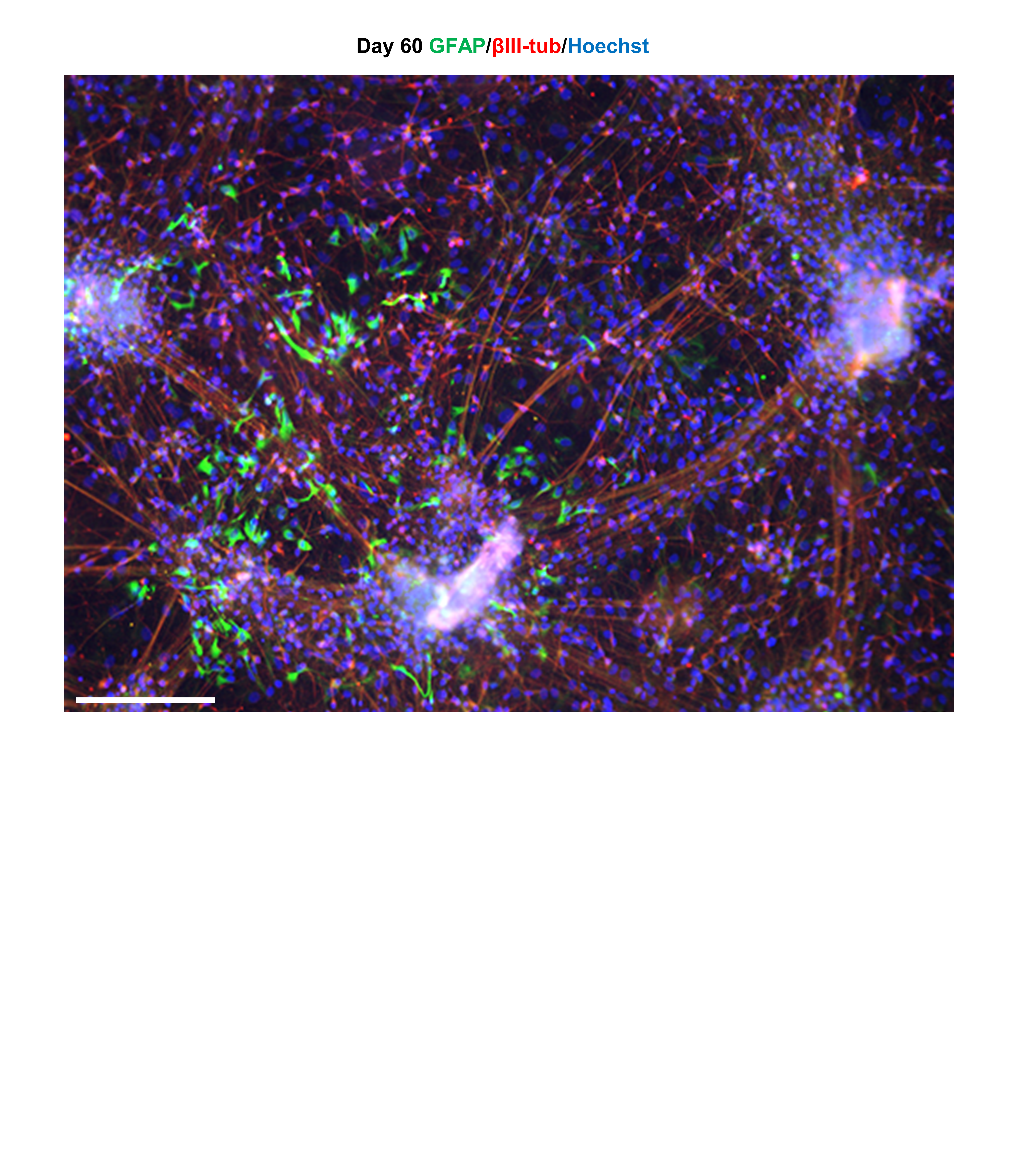

Supplement: S4 Fig — A GFAP positive subpopulation could be identified day 60. Scale bars are 200 μm. Representative images from one out of three independent neuronal differentiations. (TIF) [file pone.0261536.s005.tif]

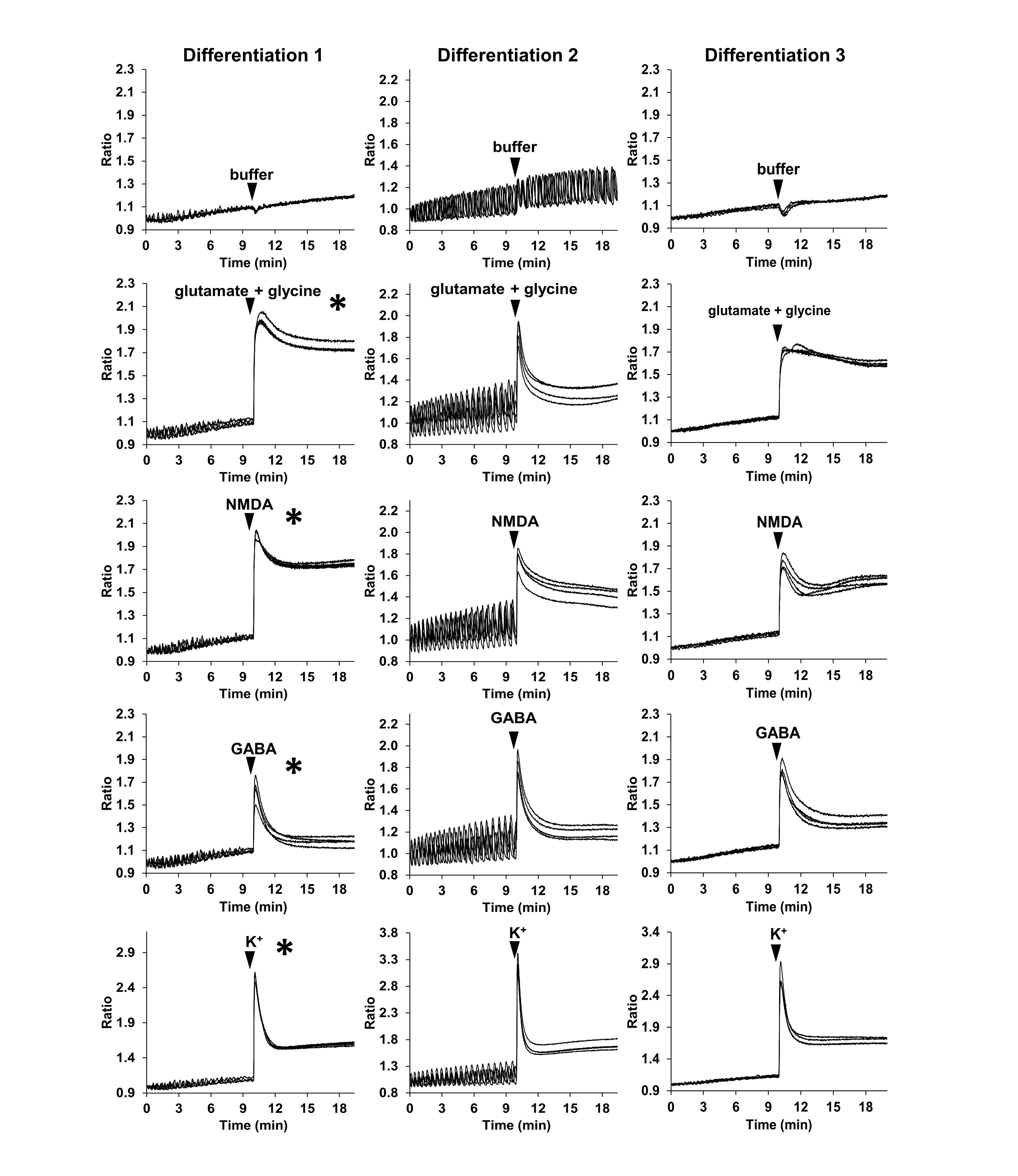

Supplement: S5 Fig — Changes in intracellular calcium in day 52–53 neurons following application of buffer control and the neurotransmitters, glutamate (300 μM glutamate +10 μM glycine), NMDA (40 μM) and GABA (100 μM), or depolarization of the membrane potential by addition of extracellular potassium chloride (25 mM). Four of the graphs (marked with a star) are identical to those in Fig 1F. The three neuronal differentiations (diff. 1, 2 and 3) are independent. (TIF) [file pone.0261536.s006.tif]

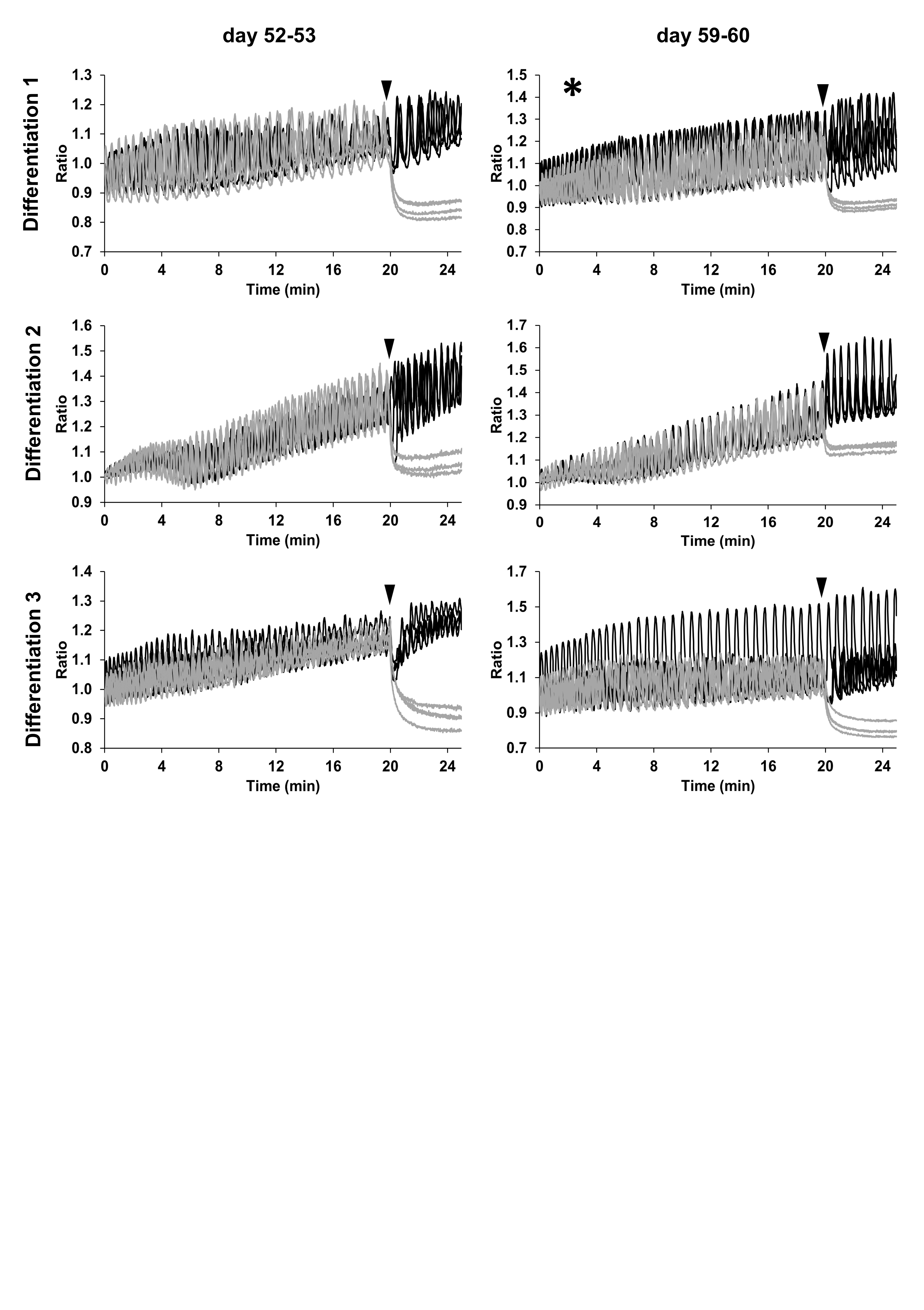

Supplement: S6 Fig — Spontaneous oscillations in day 52/53 and 59/60 neurons are eliminated by addition (timepoint marked with triangle) of 1 μM tetrodotoxin (grey traces) but not buffer (black traces). One of the graphs (marked with a star) is identical to those in Fig 1G. The three neuronal differentiations (diff. 1, 2 and 3) are independent. (TIF) [file pone.0261536.s007.tif]

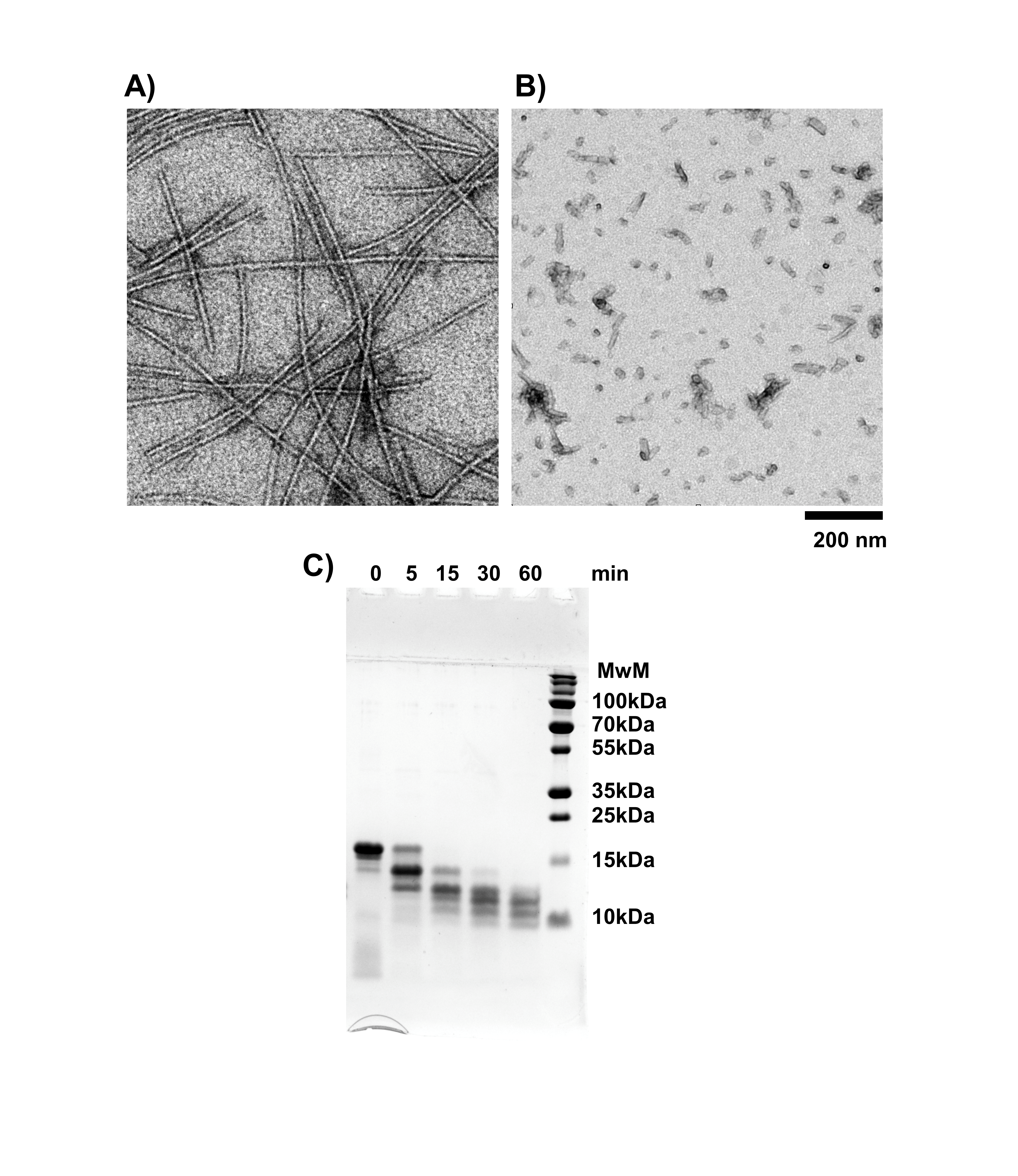

Supplement: S7 Fig — Transmission electron microscopy images before (A) and after fragmentation (B), scale bar: 200nm. (C) Proteinase K (PK, 3.8 μg/mL) degradation patterns of alpha-synuclein fibrils 91 (100μM monomer concentration) monitored over time on Coomassie stained SDS–PAGE (15%). Time (min) and molecular weight markers (MW, kDa) are shown on the top and left sides of the gels, respectively. (TIF) [file pone.0261536.s008.tif]
